# Exploring the feasibility of liquid fuel synthesis from CO_2_ under cold plasma discharge: role of plasma discharge in binary metal oxide surface modification†

**DOI:** 10.1039/d1ra04852j

**Published:** 2021-08-16

**Authors:** Nitesh Joshi, L. Sivachandiran

**Affiliations:** a Laboratory of Plasma Chemistry and Physics (LPCP), Department of Chemistry, Faculty of Engineering and Technology, SRM Institute of Science and Technology SRM Nagar, Kattankulathur Chennai-603203 India sivachal@srmist.edu.in

## Abstract

The conversion of CO_2_ to CH_3_OH over binary mixed metal oxides of NiO–Fe_2_O_3_ is investigated in the study. A series of catalysts, *i.e.*, NiO, Fe_2_O_3_, 5% NiO–Fe_2_O_3_ (5NF), 10% NiO–Fe_2_O_3_ (10NF), and 15% NiO–Fe_2_O_3_ (15NF), was tested for CO_2_ conversion and CH_3_OH selectivity performance. The results show that binary mixed metal oxides are more active in comparison to pure metal oxides. Moreover, increasing NiO mixing leads to the agglomeration of NiO particles. At 200 °C, around 1.5%, 2%, and 3.2% CO_2_ conversion is achieved for 5NF, 10NF, and 15NF, respectively. Interestingly, when cold plasma was ignited at 200 °C, around 5.4%, 6.2%, and 10.2% CO_2_ conversion was achieved for the 5NF, 10NF, and 15NF catalysts, respectively. 15NF exhibited the highest CO_2_ conversion, but produced only CH_4_. Plasma coupling with the catalyst led to an increase in the CH_3_OH yield, and around an 5.8-fold enhancement was achieved with 10NF at 200 °C compared to thermal catalysis. We showed that the combination of plasma and thermal heating brings about significant changes to the catalyst morphology, which significantly improved the catalytic activity. X-ray diffraction (XRD) and X-ray photoelectron spectroscopy (XPS) characterization revealed that plasma treatment leads to the formation of a mixture of spinel compounds (NiO–Fe_2_O_3_, NiFe_2_O_4_, and Fe_3_O_4_).

## Abstract 2

Mechanistic understanding of CO_2_ conversion to CH_3_OH over binary mixed metal oxides of NiO–Fe_2_O_3_.

## Graphical abstract

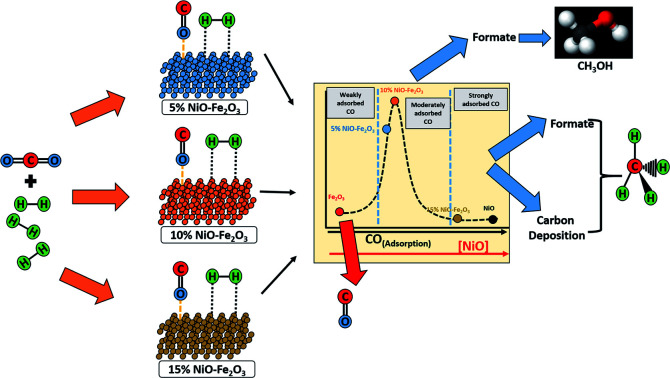

## 1. Introduction

The rising CO_2_ concentration in the atmosphere is a serious situation. Currently, the adverse effects triggered by CO_2_ emissions are being well documented and are causing global concern.^1–3^ There are various methodologies available for controlling rising CO_2_ levels. Among the viable solutions, great efforts have been made in terms of two strategies: (1) CO_2_ capture and storage^4,5^ (2) CO_2_ chemical recycling.^6,7^ It has been reported that the recycling of CO_2_ as a carbon source for value-added chemical production, such as methane (CH_4_),^8^ methanol (CH_3_OH)^9^ or polycarbonate,^10^ would be considered to be a more sustainable use of global carbon resources, which would lead to lower consumption of fossil fuels and subsequent CO_2_ emissions.^11,12^ Among the different alternatives, one of the widely investigated processes is the hydrogenation of CO_2_ to CH_3_OH (eqn (1)) using H_2_ produced from renewable energy sources.

CH_3_OH is one of the most important feedstocks as it can be used directly as a fuel, as an energy carrier in fuel cells, or as a raw material to synthesis formaldehyde, acetic acid, and olefins.^11^ CH_3_OH is traditionally produced from syngas, however, for economic and environmental reasons, there is great interest in developing a process where it is produced from the greenhouse gas CO_2_. The main problem that is currently hindering the wider application of the latter process is low CH_3_OH selectivity due to the occurrence of the competitive reverse water–gas shift (RWGS) reaction (eqn (2)).^13^ Thus, it is important to develop catalytic materials characterized by high activity and selectivity towards CH_3_OH.

The state-of-the-art catalyst for CH_3_OH synthesis is CuO/ZnO/Al_2_O_3_ (CZA), where CuO and ZnO catalyse both the CH_3_OH formation and the RWGS reaction.^13^ The reaction is catalysed at 513–533 K under 50–100 bar pressure.^13^ The commercial catalyst CZA losses its activity after several catalytic cycles due to surface poisoning and particle sintering. Additionally, to catalyse a reaction *via* thermal catalysis, a high operating temperature and pressure are applied to overcome kinetic and thermodynamic barriers.^9,14^ Thus, the industrialization of the CH_3_OH production requires an efficient catalyst with a low activation temperature.

The impact of metal ions in bimetallic catalysts is crucial, as demonstrated by Ren *et al.*,^15^ where in their study Ni–M/ZrO_2_ catalysts exhibited different product distributions with different metal ions. Iron (Fe)-based catalytic systems have been widely investigated, due to their low cost and high activity, for RWGS and Fischer Tropsch (FT) reactions to synthesis olefinic products.^11^ Gasoline fractions have been reported to be produced by Wei *et al.*^16^ using a heterogeneous catalytic system of Na–Fe_3_O_4_/HZSM-5. Herein, a CO_2_ hydrogenation reaction was initiated at a H_2_/CO_2_ ratio = 3, 320 °C, 2 MPa pressure and ∼67 mL min^−1^ feed flow rate. The authors obtained gasoline (C5 to C11 hydrocarbons with 78% selectivity) from this setup. In another study, Zhou *et al.*^17^ employed ZnO/ZrO_2_ integrated over H-ZSM5 for aromatic synthesis from CO_2_. The authors obtained 16% CO_2_ conversion and 76% selectivity towards aromatics at 340 °C under 40 bar pressure. Additionally, there are some state-of-the-art reviews available that comprehensively summarize the recent advancements in heterogeneous catalysis for CO_2_ hydrogenation to value added products.^18,19^

There are few reports available on CO_2_ reduction using mixed oxides under plasma discharge. CO_2_ conversion to CH_4_ has been carried out using NiO–Fe_2_O_3_ layered double hydroxides by Wierzbicki *et al.*^20^ Around 75% CO_2_ conversion and 100% CH_4_ selectivity were obtained using a Ni_20_Fe_1.5_ catalyst at 250 °C and 11.7 W input power. In another study, CO_2_ conversion to syngas using a Ni/Al_2_O_3_ catalyst was carried out by Ma *et al.*^21^ using plasma. The authors applied 55.4 J cm^−3^ of specific input energy and around 14% CO_2_ conversion was achieved, with CO being the main product quantified at the reactor outlet. Jwa *et al.*^22^ carried out CO and CO_2_ conversion to CH_4_ over a Ni–zeolite catalyst. The authors studied the influence of external heating on the activity of the catalysts. At a temperature of 180–360 °C, the CO_2_ conversion was <15%. Upon combining thermal heating with plasma discharge on the catalyst bed, the CO_2_ conversion increased to >95%. This enhancement is the result of the reactive species generated in the plasma reducing the catalyst activation energy barrier and thermal heating facilitating their interaction.

When it comes to the activation of catalysts and the conversion of reactants under ambient conditions, non-thermal plasma (NTP) aided catalysis is a fascinating avenue. NTP is efficient for activating CO_2_ at room temperature under atmospheric pressure conditions.^23,24^ The NTP process of catalysis operates *via* a completely different mechanism of electron impact dissociation, thus, it consumes less energy compared to thermal catalysis. In thermal catalysis, the vibrational excitation of molecules is a key factor that requires higher energy and therefore impacts the overall energy efficiency.^25^ Various types of NTPs have been employed in the successful conversion of CO_2_ to CO or CH_4_ or oxygenates.^14,26–30^ The dielectric barrier discharge (DBD) is one such NTP that has been widely studied, as it has a simplistic design and is operational under ambient conditions. Thus, it could be of prime importance from a commercialisation point of view. In terms of DBD plasma, only a handful of studies have focused on the conversion of CO_2_ to oxygenate. For instance, Bill *et al.*^31^ showed around 0.2% CH_3_OH yield by using 400 W input power at a 250 mL min^−1^ feed flow rate. Interestingly, the commercial CH_3_OH catalyst Cu/ZnO/Al_2_O_3_ under atmospheric pressure shows 12% CO_2_ conversion and a CH_3_OH selectivity of 0.4%, but upon increasing pressure to the 8 bar, the conversion rose to 14% with 10% CH_3_OH selectivity.^32^ Enhanced CO_2_ conversion to CH_3_OH was reported by Wang *et al.*^24^ The authors showed that CH_3_OH selectivity could be increased to 53.7% with 11% yield using Cu/γ-Al_2_O_3_ at room temperature under atmospheric pressure. More recently, a Co_*x*_O_*x*_/MgO catalyst system was employed for the conversion of CO_2_ to CH_3_OH at room temperature under atmospheric conditions.^33^ The authors observed that 33% CO_2_ conversion and 31% CH_3_OH selectivity were achieved using 10 W of input power.

In our previous work, we successfully showed that binary mixed metal oxides are more active towards CO_2_ conversion and CH_3_OH production.^34^ The main aim of this work was to employ mixed binary metal oxides of NiO–Fe_2_O_3_ for the hydrogenation of CO_2_ to CH_3_OH using non-thermal plasma. Dedicated efforts were made to investigate (i) the influence of individual and binary mixed metal oxides on CO_2_ conversion and CH_3_OH production, (ii) the impact of NiO mixing on Fe_2_O_3_ for CO_2_ conversion and CH_3_OH selectivity, (iii) understanding the role that plasma power and operating temperature have on CO_2_ conversion and products distribution, and (iv) the effect that plasma discharge has on catalyst surface modification.

1 CO_2_ + 3H_2_ → CH_3_OH + H_2_O, Δ*H*^0^_25°C_ = −49.5 kJ mol^−1^

2 CO_2_ + H_2_ ↔ CO + H_2_O, Δ*H*^0^_25°C_ = 41 kJ mol^−1^

## 2. Experimental description

### 2.1. Preparation of the catalyst

In this study, the amount of NiO was varied: 5%, 10% and 15% on Fe_2_O_3_ and labelled as 5NF, 10NF and 15NF, respectively. The detailed NiO–Fe_2_O_3_ catalyst synthesis procedure has been reported elsewhere.^31^ Briefly, various amounts of Ni(NO_3_)_2_·6H_2_O (99% pure) were mixed with Fe(NO_3_)_3_·9H_2_O (99% pure) and dissolved in 50 mL of ethanol (96% pure) and a homogeneous solution was made with constant stirring. Then, the precursor was precipitated as hydroxide sol using 10% NH_4_OH solution at a flow rate of 1 mL min^−1^. At pH ∼ 12, complete precipitation was reached, then the sol was aged at 85 °C overnight. Then the precipitate was centrifuged-washed several times using distilled water to remove the impurities. The washed residue was dried at 100 °C (for 2 h) and then calcined at 600 °C for 3 h.

### 2.2. Catalyst characterization

The synthesised and used catalysts were characterised by different techniques. X-ray diffraction (XRD) data were obtained using a PANalytical X'pert3 instrument [Cu Kα 1.54 Å, 40 kV, 40 mA]. The catalyst surface morphology was studied using nanoscale images obtained using a high-resolution field emission electron microscopy (FESEM, Quanta 200) and high-resolution transmission electron microscopy (HRTEM JEOL, Japan) instruments. Along with electron images, energy-dispersive X-ray (EDX) spectroscopy data was obtained to determine the elemental composition, which is provided in Fig. S1a–c in the ESI.† The elemental mapping image of 10NF is provided in Fig. S1d in the ESI,† which was obtained using FESEM. X-ray photoelectron spectroscopy (XPS) analysis was carried out using a PHI Versaprobe III to obtain further information on the elemental composition and oxidation state of the catalysts. The physical properties of the catalysts, *i.e.*, the total surface area (Brunauer–Emmett–Teller, BET) was measured using an Autosorb iQ Station at 77 K by N_2_ physisorption. Before the analysis, the catalysts were degassed under a He atmosphere at 150 °C for 3 h. The data from the N_2_ sorption study is provided in Fig. S2 in the ESI.† The Lewis and Brønsted acidic sites were determined using Fourier-transform infrared (FTIR) spectroscopy (SHIMADZU IR TRACER-100) by adsorbing pyridine. 10 mg of pyridine adsorbed catalysts were thoroughly mixed with 100 mg of NaCl to maintaining sample uniformity and were then analysed by FTIR spectroscopy in attenuated total reflectance (ATR) mode. The FTIR analysis is reported in Fig. S3 in the ESI.†

### 2.3. Plasma reactor and experimental setup

**Fig. 1:**
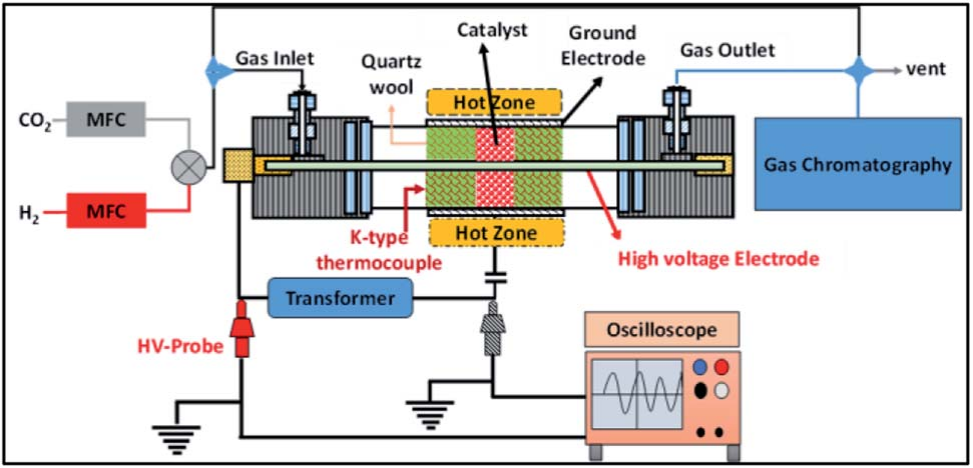
A schematic diagram of the experimental setup.

The experimental setup employed in this study is shown in Fig. 1. A detailed description of the reactor has been reported elsewhere.^31^ Briefly, in this setup the cylindrical quartz tube is 600 mm in length, the outer diameter (OD) of the tube is 25 mm, the wall thickness of the tube is 3 mm and the inner diameter (ID) of the reactor is 19 mm. A 12 mm (OD) stainless steel rod is used as an inner electrode, which leads to a 3.5 mm discharge gap. In the experiments, the discharge length was fixed at 100 mm by wrapping the quartz tube in stainless steel mesh. The plasma was generated using a step-up transformer supplied by Jayanti Transformer (Chennai, India). The AC voltage was varied from 10 to 18 kV at a constant frequency of 50 Hz.

The electrical parameters were calculated using two high voltage probes with 1 : 100 attenuation (Zeal Manufacturing Service Limited, Pune, India), which were connected to an oscilloscope (Keysight, 70 MHz 2Ga s^−1^) as shown in Fig. 1. The power dissipated into the reactor was calculated using the Lissajous method.^35,36^

For catalytic CO_2_ conversion, the binary metal oxides (0.5 g) were placed in the centre of the quartz tube and quartz wool (QW 3.67 g) was used to sandwich the catalyst. The catalyst packing mode used in this work is different from that in our previously reported work.^31^ In the previous study, the catalyst was loaded on QW using a dip-coating method. However, in this study, due to practical issues related to catalyst characterisation, before and after plasma treatment, the catalyst was packed in sandwich mode. The CO_2_ and H_2_ composition was set to a ratio of 1 : 3 and the total flow rate was fixed at 100 mL min^−1^. The discharge volume was fixed to 17.4 cm^3^.

The feed flow rate through the reactor was fixed to 100 mL min^−1^ unless otherwise mentioned, and H_2_ and CO_2_ were mixed in a 3 : 1 ratio. Commercial-grade CO_2_ (99.5%), H_2_ (99.99%), N_2_ (99.999%) and zero air (99.999%) were supplied by Rana Industrial gases (Chennai, India). The zero air was used for catalyst pre-treatment and N_2_ was used as a dilutant gas during gas chromatography (GC) calibration. The CO_2_ and H_2_ flow were regulated using the respective calibrated mass flow controllers (MFC, KOFLOC, Japan).

### 2.4. Methodology and product analysis

The gaseous products at the reactor outlet were analysed using gas chromatography (GC, Perkin Elmer-Claraus 580) equipped with an online gas sampling loop (2 mL). A thermal conductivity detector connected to a ShinCarbon ST Column (mesh size of 100/120, length of 2 m and an inner diameter of 1/11th inch) was used for eluting the reactants and products. The reaction output, such as CO_2_ conversion, product selectivity and product yield, was calculated using the equations provided in the ESI.†

### 2.5. Experimental procedure

#### 2.5.1. Thermal catalysis

As shown in Fig. 1, a K-type thermocouple placed inside the reactor, on QW, was used to monitor the catalyst temperature. For all of the experiments, a fresh catalyst was used, and before each experiment, the catalyst was pre-treated under zero air at 300 °C for 1 h. Then, the reactor was cooled to room temperature. At room temperature, the feed gas was sent to the reactor and the blank concentrations of CO_2_ and H_2_ were obtained after reaching the adsorption/desorption equilibrium. To understand the effect of temperature on the CO_2_ conversion and CH_3_OH selectivity, the catalyst bed temperature was varied between 100 and 250 °C. Online sampling was carried out after 60 min of reaching the set temperature (steady state). For each set temperature, a minimum of three samples were analysed to ensure reproducibility and each experiment was repeated a minimum of three times.

#### 2.5.2. Plasma and thermal-plasma catalysis

For the thermal-plasma catalytic process, the plasma discharge was ignited after reaching the thermal catalytic steady state (about 30 min of the thermal catalytic process at the set temperature). The online sampling was performed after 10 min of continuous plasma treatment. It is noteworthy to mention that during plasma treatment the furnace was switched off to avoid electrical perturbation. However, the temperature difference before and after plasma treatment was around ±10 °C.

## 3. Results and discussion

### 3.1. Physiochemical characterization of the catalysts

**Fig. 2:**
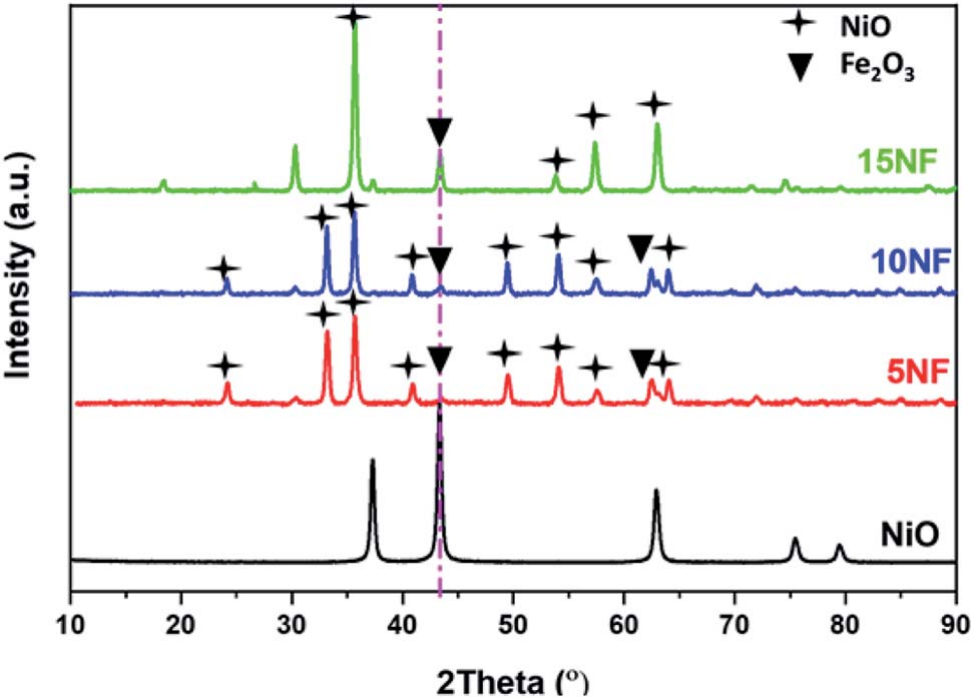
XRD characterization of the synthesised catalysts.

The XRD patterns of the synthesised catalysts are shown in Fig. 2. The XRD patterns show that the synthesized catalysts are pure and crystalline. The diffraction peaks at 37.3, 43.3, 62.9 and 75.3 confirm the presence of NiO, which correspond to the (111), (200), (220), and (311) planes, respectively. For NiO–Fe_2_O_3_ binary mixed oxides, the characteristic peak for NiO, centred at 43.3°, is used for calculating the particle size, whereas the intense peak at 33° is used to calculate the particle size of Fe_2_O_3_. The details of particle size calculations using the Scherrer equation for all of the catalysts are presented in Table 1. It can be observed that the particle size of NiO increases upon increasing the % of metal oxide mixing. This is due to the weak interaction between NiO and Fe_2_O_3_, which does not suppress the agglomeration and formation of large NiO clusters unlike on other supports, such as porous Al_2_O_3_, which interacts strongly with active metals to suppress particle growth.^37,38^ However, as compared in Table 1, the Fe_2_O_3_ particle size was not significantly affected due to the dopant action of NiO.

**Table 1:** Physicochemical properties of the various catalysts

Catalyst	Particle size (nm)		Metal mixing^b^ (%)	*S*_BET_ c (m^2^ g^−1^)	Pore volume (cm^3^ g^−1^)	Pore size (Å)
	NiO	Fe_2_O_3_				
Fe_2_O_3_	—	32	—	NA	NA	NA
NiO	11	—	—	NA	NA	NA
5NF	40.8	20.4	5.2	23.5	0.15	195.6
10NF	178	29.9	10.3	9.1	0.03	28.5
15NF	8934	25.7^a^	18.7	3.6	0.07	13.8

a The ICCD database reference no 00-039-1346 peak at the highest intensity at 35.631 was used.

b Results based on EDX analysis.

c The specific surface area obtained from BET measurements.

**Fig. 3:**
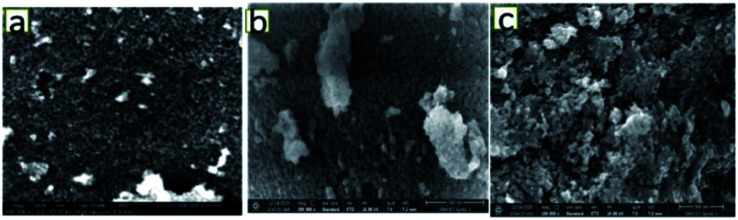
FESEM images of (a) 5% NiO–Fe_2_O_3_, (b) 10% NiO–Fe_2_O_3_, and (c) 15% NiO–Fe_2_O_3_.

Fig. 3 shows the FESEM images of the various catalysts. As can be seen from Fig. 3(a), on 5NF the NiO particles are uniformly distributed, on the other hand for 10NF and 15NF the NiO particles are agglomerated upon increasing the amount of metal mixing, as can be seen from Fig. 3(b) and (c), respectively. It should be noted that the particle size calculated from the XRD data (using the Scherrer equation) is in good agreement with what is observed in the SEM images. It can be observed that, on the one hand, the increase in NiO mixing leads to an increase in particle size due to agglomeration. On the other hand, an increase in metal mixing decreases the surface area and pore volume, as can be seen from Table 1. The decrease in the total surface area can be attributed to the agglomeration of NiO particles on the Fe_2_O_3_ support, which is in line with the particle size calculated using XRD.

### 3.2. Plasma energy deposition into the packed bed reactor

**Fig. 4:**
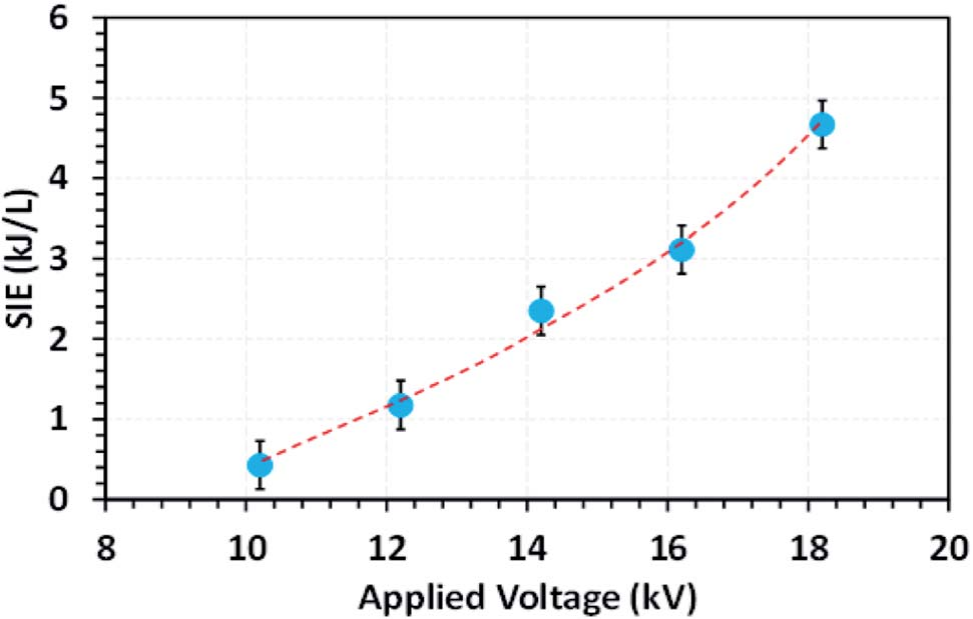
The evolution of the SIE as a function of the applied voltage (feed flow rate: 100 mL min^−1^, CO_2_/H_2_ = 1 : 3, frequency: 50 Hz).

Fig. 4 shows the effect of applied voltage on the specific input energy (SIE) delivered into the reactor packed with a 10NF catalyst, wherein it can be seen that the increase in applied voltage exponentially increases the SIE. It is worth mentioning that no significant difference in SIE was observed for the different catalyst packed reactors. The increase in applied voltage increases the charge build-up across the dielectric material and thus increase the power injected into the system. Therefore, it can be proposed that the enhanced charge improves the CO_2_ conversion under similar operating conditions. The detailed plasma diagnostics were evaluated and the results are shown in Fig. S4 in the ESI.†

### 3.3. Thermal catalysis study

**Fig. 5:**
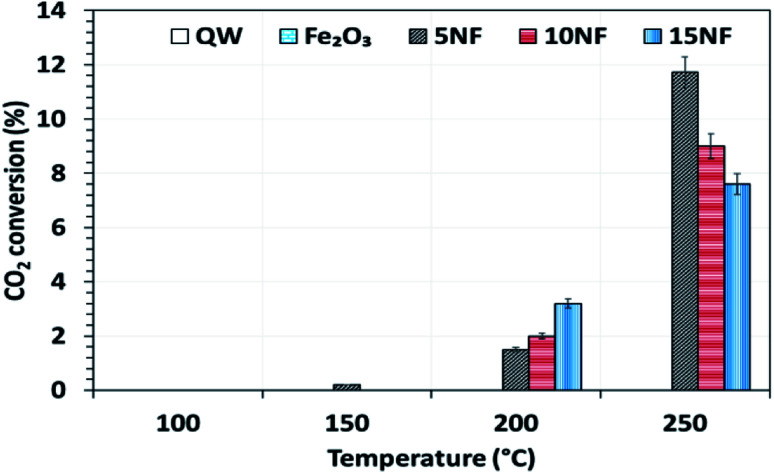
The influence of temperature on CO_2_ conversion for the different catalysts. Reaction conditions: catalyst, 0.5 g; H_2_/CO_2_ = 75/25, 100 mL min; *P* = 1 atm.

The catalyst bed temperature was linearly varied from 30 to 250 °C and the CO_2_ conversion at different temperatures is shown in Fig. 5. We observed that for all of the catalysts the CO_2_ conversion increases with an increase in temperature. Moreover, even at 250 °C the pure metal oxides *i.e.* NiO and Fe_2_O_3_ exhibited around 3.5% and <1% CO_2_ conversion, respectively (results not reported in the figure). On the one hand, at 200 °C the CO_2_ conversion increased from 1.7 to 3.7% upon increasing the NiO mixing from 5 to 15%. On the other hand, at 250 °C the mixing of 5% NiO with Fe_2_O_3_ improved the CO_2_ conversion by three-fold (11.7%) under similar operating conditions. However, a further increase in the amount of NiO mixing exhibited a negative effect, *i.e.*, decreased the CO_2_ conversion to 9% (10% NiO) and 7.5% (15% NiO). This decrease in conversion with an increase in NiO mixing could be attributed to the (i) NiO particle size and (ii) the oxidation of products such as CH_4_ and CH_3_OH to CO_2_ at high temperatures. As reported in Table 1, the NiO particle size increased significantly with an increase in the amount of NiO mixing. The bigger the NiO particle size the more difficult it is to reduce compared to small sized NiO particles, as hydrogen mass transfer is hindered.^34^

3 2CO_(ads)_ → C_(ads)_ + CO_2_

4 C_(ads)_ + 2H_2(ads)_ → CH_4_

**Fig. 6:**
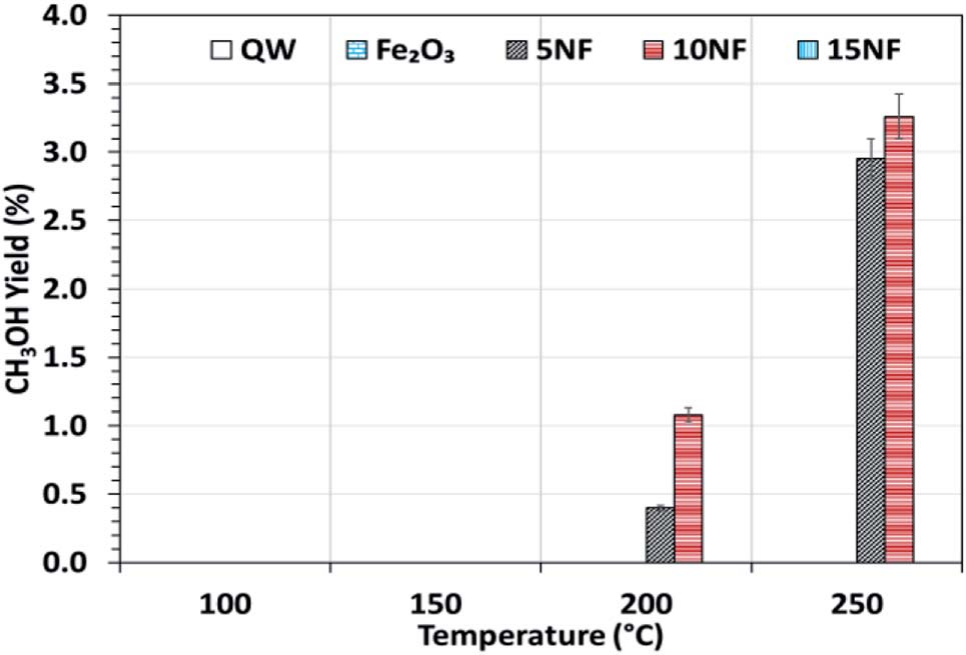
CH_3_OH yield as a function of the reaction temperature. Reaction conditions: catalyst, 0.5 g; H_2_/CO_2_ = 75/25, flow rate, 100 mL min; *P* = 1 atm.

Fig. 6 shows the CH_3_OH yield as a function of temperature varied between 100 and 250 °C. It can be observed that the CH_3_OH yield increases with an increase in temperature. At 200 °C, the increase in NiO mixing from 5% to 10% increases the CH_3_OH yield from 0.4 to 1%, respectively. At 250 °C, a maximum yield of 3.2% of CH_3_OH was obtained for the 10NF catalyst. However, a further increase in the amount of NiO to 15% exhibited no CH_3_OH formation. From the XRD results, it can be observed that the increase in NiO mixing (15NF) leads to the formation of NiFe_2_O_4_ and large particle size. Thus, it is suggested that spinel structures and larger NiO particles do not favour CH_3_OH formation in thermal catalysis. This could be due to weaker and/or no chemisorption of CO and H_2_ on spinels, as evidenced by Zhang *et al.*^39^ The authors synthesised Ni/α-Al_2_O for CO_2_ hydrogenation to CH_4_. It was evident that nickel aluminate spinel structures (NiAl_2_O_4_) were formed on the catalyst surface, which was confirmed by XRD. NiAl_2_O_4_ is inactive towards the chemisorption of H_2_ and CO and thus CH_4_ is the main product quantified. Thus, it is thought that the species required for CH_3_OH does not bind to the 15NF catalyst surface. Indeed, during CH_3_OH synthesis the binding energy of intermediates (namely CO, formate others) is of prime importance as it determines the nature of the product. As reported in Fig. S1 in the ESI,† the pyridine adsorption study revealed that an increase in the amount of NiO also increased the number of Lewis basic sites. Therefore, the CO_2_ adsorption on the catalyst surface also increases and more CO_2_ conversion is expected, however, it should also be noted that the nature of the product is dependent on the intermediate binding strength. If intermediates (CO, formate) are strongly adsorbed on the NiO surface then this leads to CH_4_ formation, *i.e.*, the Boudouard reaction is dominant, as reported in eqn (3) and (4).

By mixing metal oxides with different binding strengths toward the intermediates (CO and formate), a binary metal oxide mixture with optimum binding energy for the intermediates could be prepared. The optimum binding energy means that the intermediates are neither adsorbed strongly nor loosely on the catalyst surface. On the one hand, as we stated above, if the intermediates (CO and formate), adsorb strongly on NiO this leads to CH_4_ formation, on the other hand, if CO adsorbs weakly (for example on Fe_2_O_3_), it is easily desorbed into the gas phase. Indeed, both sets of conditions significantly decrease the CH_3_OH production. Thus, it is proposed that 10NF shows the optimum binding energy with the intermediates and thus the highest yield of CH_3_OH.

**Fig. 7:**
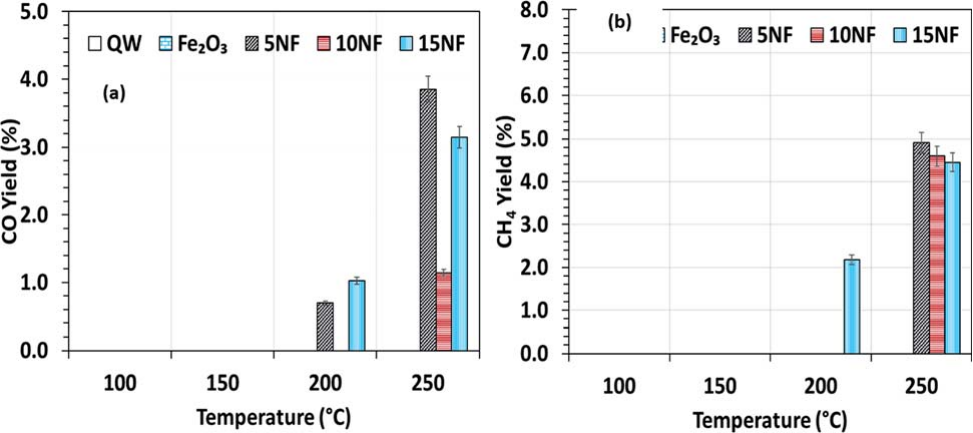
(a) CO yield and (b) CH_4_ yield as a function of the catalyst bed temperature.

To investigate the role that the amount of NiO has on CO and CH_4_ formation, the respective yields were calculated as a function of temperature and the results are reported in Fig. 7(a) and (b). We observed that at 200 °C, the CO yield increased from 0.7% to 1.2% upon increasing the NiO content from 5% to 15%. Notably, with the 10NF catalyst, CO was not quantified at the reactor downstream. This result supports our hypothesis that all of the CO adsorbed with the optimum binding energy on the 10NF catalyst is converted to CH_3_OH. Moreover, at 250 °C, the CO yield decreases with an increase in NiO mixing. It should also be noted that 10NF exhibits the lowest yield of CO, nevertheless, it exhibits the highest yield of CH_3_OH (Fig. 6). These findings provide evidence for the fact that CO is converted into CH_3_OH on the catalyst surface.

As can be seen in Fig. 7(b), only 15NF exhibits around 2.1% CH_4_ yield at 200 °C. Moreover, it is noted that with an increase in the reaction temperature the CH_4_ yield also increases.

Remarkably, at 250 °C, the CH_4_ yield decreases with an increase in the amount of NiO. This CH_4_ formation can be correlated to a high-temperature Boudouard reaction, *i.e.*, hydrogenation of carbon (C) to CH_4_. Furthermore, the decrease in the CH_4_ yield can be attributed to the oxidation of CH_4_ to CO_2_, thus decreasing the net CO_2_ conversion.

### 3.4. Effect of plasma discharge on the CO_2_ conversion and CH_3_OH yield

**Fig. 8:**
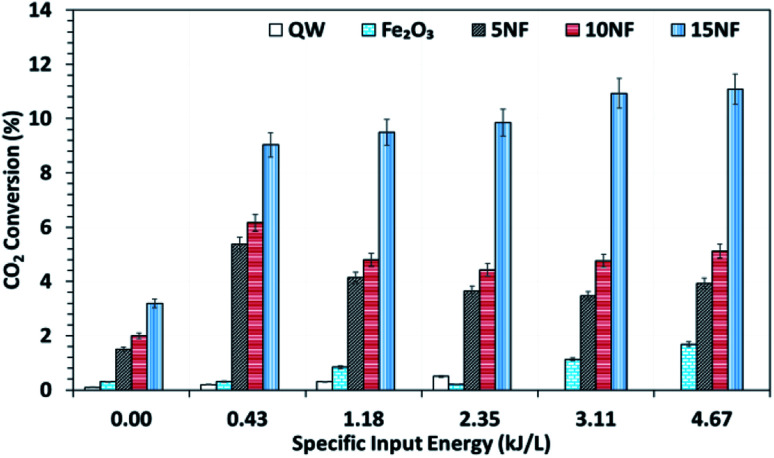
CO_2_ conversion at 200 °C as a function of the SIE. The SIE was varied between 0.43 and 4.67 kJ L^−1^*via* changing the applied voltage at a fixed frequency of 50 Hz.

**Fig. 9:**
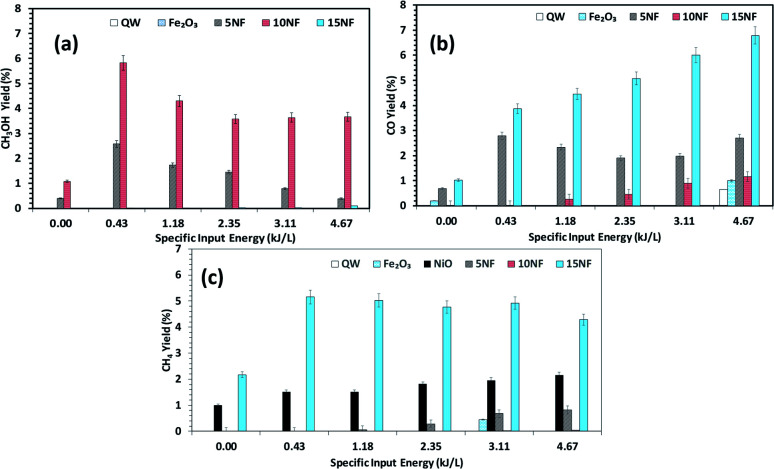
Effects of the SIE on the product distribution at 200 °C. The yield of (a) CH_3_OH, (b) CO, and (c) CH_4_. Experimental conditions: catalyst, 0.5 g; H_2_ : CO _2_, 3 : 1; total flow rate, 100 mL min^−1^.

It should be noted that at room temperature the plasma discharge does not show any CO_2_ conversion. On the one hand, up to 150 °C, the plasma-thermal catalytic process exhibits less than 1% CO_2_ conversion. On the other hand, at 250 °C, despite around 10% CO_2_ conversion, CH_4_ was the major product in the thermal catalytic process. Moreover, we also found that when plasma discharge was ignited above 14 kV (2.6 kJ L^−1^) at 250 °C, owing to the exothermic reaction and plasma injected power, the quartz reactor was broken (repeated five times with a similar result observed). Therefore, the effect of plasma discharge on CO_2_ conversion and product distribution was investigated only at 200 °C and the results are reported in Fig. 8 and 9 as a function of specific input energy (SIE).

As showed in Fig. 8, without plasma discharge (denoted by 0.00 kJ L^−1^), the CO_2_ conversions were 1.5%, 2%, and 3.2% for 5NF, 10NF and 15NF, respectively. Interestingly, when plasma discharge was added (SIE of 0.43 kJ L^−1^, 0.8 W, 10.2 kV) the conversions increased to 5.4%, 6.2%, 10.2% for 5NF, 10NF and 15NF, respectively. Indeed, the three-fold increase in CO_2_ conversion could be due to the synergism between the plasma discharge and the catalyst.^30^ The plasma discharge along with thermal catalysis leads to changes in discharge behaviour and the plasma parameters can be found in Fig. S4–S8 in the ESI.† The inductive effect of heating and applying plasma is crucial to increasing the effective capacitance by virtue of charge separation. Remarkably, the CO_2_ conversion decreases upon an increase in the SIE. For example, when SIE was increased from 0.43 to 4.67 kJ L^−1^ the CO_2_ conversion decreased from 5.4% to 3.9% and 6.2% to 5.1% for the 5NF and 10NF catalysts, respectively. However, for the 15NF catalyst, the CO_2_ conversion increased from 9% to 11% with increasing SIE. The decrease in CO_2_ conversion for the 5NF and 10NF catalysts can be attributed to the water gas shift reaction (WGSR), as reported in eqn (5). The Ni-based catalyst is very active towards the WGSR, and the CO molecules are converted into CO_2_.^18,40^5
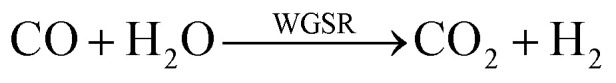


Fig. 9(a)–(c) shows a comparison of the yields of the major products quantified at the reactor downstream as a function of SIE at 200 °C. We evidenced that, similar to CO_2_ conversion, the CH_3_OH yield also increased when the plasma discharge was ignited. This finding emphasises the fact that plasma discharge activates the catalyst surface at low temperatures and facilitates the liquid fuel CH_3_OH production. As can be seen in Fig. 9(a), around 6% CH_3_OH yield is achieved with the 10NF catalyst at 0.43 kJ L^−1^ SIE, which is six-fold higher than the yield obtained under similar operating conditions in the absence of plasma. However, an increase in SIE had a negative effect on the CH_3_OH yield for both the 5NF and 10NF catalysts. The CH_3_OH yield decreases with increasing SIE, and around 4% is obtained with 4.67 kJ L^−1^ SIE. The decrease in the CH_3_OH yield can be attributed to (i) the decrease in CO_2_ conversion as demonstrated in Fig. 8 and (ii) the decomposition of the produced CH_3_OH to CO_2_. Notably, similar to the thermal catalytic process (Fig. 6) CH_3_OH was not produced with the 15NF catalyst for all of the investigated SIE values. This can be ascribed to the weak/absence of chemisorption of CO and H_2_ to produce CH_3_OH.

As reported in Fig. 9(b), at 200 °C the plasma discharge significantly improves the CO yield for all of the studied catalysts. Indeed, with the 15NF catalyst, the CO yield increased with increasing SIE. The enhanced CO yield can be attributed to the improved CO_2_ conversion, as shown in Fig. 8. Thus, it can be proposed that the inverse spinel NiFe_2_O_4_ assists the CO_2_ splitting into CO rather than undergoing a hydrogenation reaction to form CH_3_OH. Remarkably, the 10NF catalyst exhibited a maximum of 1% CO yield at 4.67 kJ L^−1^, since a maximum of converted CO_2_ was hydrogenated to CH_3_OH, as shown in Fig. 9(a). As can be seen in Fig. 2, the PXRD pattern confirms that the 15NF catalyst has an inverse spinel NiFe_2_O_4_ structure (ICDD database reference number 01-086-2267). In NiFe_2_O_4_, the active metal is encapsulated in the lattice of Fe_2_O_3_ making it difficult to reduce NiO to Ni at low temperature.^41^ Moreover, the NiO particles that are not encapsulated in the crystal lattice assist the conversion of CO_2_ to CO and hydrogenation products such as CH_4_. In our previous study, we confirmed that the pure NiO catalyst selectively produces CH_4_ and CO.^31^

As shown in Fig. 9(c), only the 15NF catalyst produces a significant amount of CH_4_. We also showed that the coupling of plasma at 200 °C doubled the CH_4_ yield. Moreover, the increase in SIE does not significantly influence the CH_4_ yield. Thus, it can be proposed that when the NiO mixing reaches an optimum value only then it does facilitate the further conversion of formate (which is one of the intermediates in CO_2_ to CH_4_ conversion) into CH_4_.^35^ Therefore, the catalyst becomes more selective towards CH_4_ rather than other products such as CO and CH_3_OH. Moreover, the CH_4_ production and the reverse WGSR are competitive reactions and an increase in applied voltage facilitates CO formation, as shown in Fig. 9(b).

These findings enabled us to propose the following hypotheses: (i) at 200 °C, the plasma discharge on the catalyst significantly modifies the catalyst surface, thus the catalytic activity decreases in line with the plasma treatment time and (ii) the NiO particle size is modified by the plasma discharge. To provide evidence to prove these hypotheses, the catalysts before and after plasma treatment were characterized using PXRD, TEM and XPS techniques, with the results discussed in the following section.

## 4. Effects of plasma discharge on catalyst changes

**Fig. 10:**
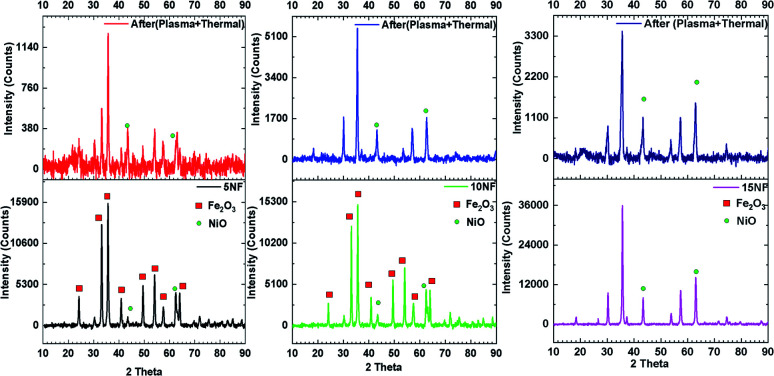
PXRD patterns of the catalyst before (bottom) and after (top) plasma treatment at 200 °C.

Fig. 10 shows a comparison of the PXRD patterns of the catalyst before and after 30 min of plasma treatment at 200 °C with 2.4 kJ L^−1^ SIE at a 1 : 3 feed ratio of CO_2_ : H_2_ (100 mL min^−1^). After plasma treatment, the 5NF and 10NF catalysts show different PXRD patterns compared to their patterns recorded as fresh catalysts. Thus, it can be concluded that the plasma discharge at low temperature brings about significant changes to the catalyst surface. We observed two important changes in the PXRD patterns: (i) the oxidation state of Fe is reduced from +3 to a mixture of +1 and +2, *i.e.* Fe_2_O_3_ to Fe_3_O_4_ and (ii) the NiO particle size is significantly reduced. Indeed, in thermal catalysis, even under reducing conditions at 200 °C, the catalyst does not show any significant changes. However, the additional energy provided by plasma discharge lowers the activation energy barrier and thus results in changes. Remarkably, in terms of the 15NF catalyst after plasma treatment, no significant modification was observed in the PXRD pattern. Moreover, the PXRD pattern of the synthesised 15NF catalyst matches an inverse spinel NiFe_2_O_4_ reference pattern from the ICDD database (reference number 01-086-2267). The NiFe_2_O_4_ is magnetic and crystallises in a face centred cubic structure. The NiFe_2_O_4_ has an inverse spinel structure, wherein Ni^2+^ occupies octahedral sites and Fe^3+^ occupies half of the tetrahedral and octahedral voids. The active metal oxide NiO is encapsulated in the lattice of Fe_2_O_3_ and it is difficult to reduce NiO under our operating conditions.^38^ After plasma treatment, the 10NF catalyst PXRD pattern exactly matches that of NiFe_2_O_4_, similar to the 15NF catalyst. However, the 10NF catalyst exhibits a higher CH_3_OH yield than 15NF.

The only difference between these two catalysts is the plasma discharge assisted inverse spinal formation, at low temperature, from the NiO–Fe_2_O_3_ mixture. Thus, the enhanced catalytic activity of 10NF can be attributed to its particle size, the nature of its surface and the mixture of the formation of inverse spinel NiFe_2_O_4_ and Fe_3_O_4_.

Table 2 shows a comparison of the impact that plasma discharge has on the particle size and lattice parameters of NiO calculated from the PXRD patterns. This is evidence that the plasma discharge on the catalyst surface significantly reduces the particle size of NiO, irrespective of the amount of mixing. The d-spacing and lattice parameters of the 10NF and 15NF catalysts were also modified by plasma discharge, as observed in the shifting of the diffraction peaks to lower 2*θ* values. The shifting of the peaks is a result of a reduction in the NiO particle size. Moreover, the plasma discharge does not affect the Fe_2_O_3_ particle size. It should also be noted that after 30 min of plasma treatment the 15NF catalyst exhibited a particle size of around 13 nm, however, CH_3_OH was not produced, as shown in Fig. 9(a). Thus, it can be concluded that rather than the particle size, the nature of the spinel structure significantly influences the CO_2_ conversion and CH_3_OH selectivity.

**Table 2:** Effects of plasma discharge on the NiO particles

Catalyst	NiO particle size (nm)		d-spacing (Å)		Lattice parameter (Å)	
	Before	After	Before	After	Before	After
5NF	40.8	13.2	3.25	3.25	6.51	6.51
10NF	178	17.6	3.14	1.97	6.28	3.95
15NF	8934	13.1	4.62	2.42	9.25	4.85

**Fig. 11:**
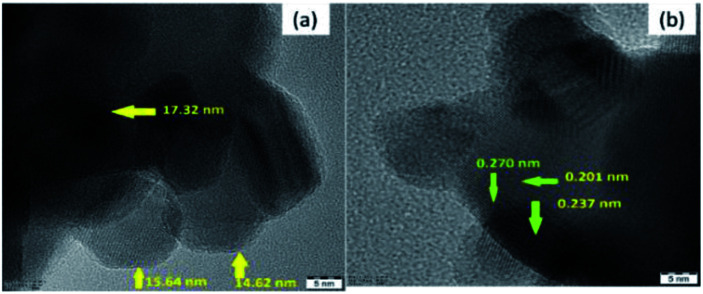
HRTEM images of the plasma-treated 10NF catalyst.

To provide evidence for the reduction in the particle size by plasma treatment, the plasma-treated 10NF catalyst was characterized by high-resolution TEM (HRTEM) and the results are shown in Fig. 11. The images reveal that the NiO particles are spherical in structure, the average particle size is 15.1 nm and the average d-spacing value is 0.228 nm (2.28 Å).

As shown in Table 2, after plasma treatment, from PXRD the calculated NiO particle size for 10NF was found to be 17.6 nm and the d-spacing was 0.197 nm (1.97 Å). The observed TEM and calculated PXRD result are in good agreement. Thus, it can be concluded that the plasma discharge at 200 °C on the powder sample significantly reduces the particle size and improves the CO_2_ conversion and CH_3_OH selectivity.

**Fig. 12:**
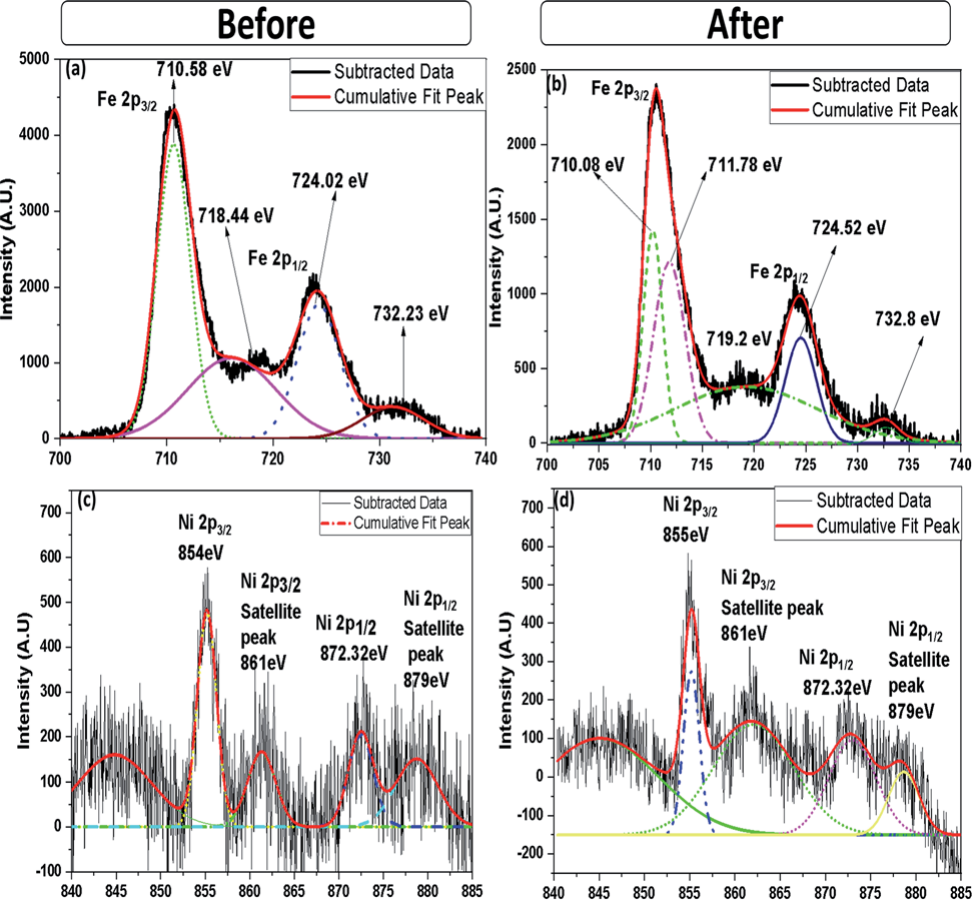
High-resolution (a) Fe 2p and (c) Ni 2p XPS spectra (before plasma treatment) and (b) Fe 2p and (d) Ni 2p XPS spectra (after plasma treatment).

XPS analysis was carried out to obtain further information on the elemental composition and oxidation state of 10% NiO–Fe_2_O_3_ (10NF) catalyst before and after plasma treatment, with the results reported in Fig. 12(a)–(f). The X-ray source of the XPS measurements was Mg Kα, and the binding energies of the Fe 2p and Ni 2p spectra were calibrated according to the binding energy of C 1s of the adventitious C at 284.5 eV. The survey spectrum, before and after plasma treatment, exhibited only Fe, Ni and O along with C peaks, moreover, no significant difference was observed. As can be seen from Fig. 12(a), before plasma treatment two distinct peaks centered at 710.58 and 724.02 eV corresponding to the Fe 2p_3/2_ and Fe 2p_1/2_ spin–orbit peaks, respectively, were observed, identifying the Fe^3+^ oxidation state on the NiO–Fe_2_O_3_ surface.^42^ Interestingly, after plasma exposure for 30 min, the peak at 710.58 eV deconvolutes into two peaks centred at 710.08 and 711.78 eV. The oxidation states affect the binding energy of the material. The peak at 710.08 eV can be attributed to Fe^2+^ and the peak at 711.78 eV can be ascribed to Fe^3+^. These findings emphasise that plasma treatment reduces Fe^3+^ partially to Fe^2+^.^39,43^ Moreover, it should be noted that before plasma treatment there are fewer intensity signals for the shakeup satellites at 732.23 and 718.44 eV. Furthermore, after plasma treatment the intensity slightly increased and the peaks moved to higher binding energies (719.2 and 732.8 eV) indicating the presence of a separate hematite phase in the sample.

The Ni 2p spectra, before and after plasma treatment, are compared in Fig. 12(c) and (d). Before plasma treatment the sample exhibits two spin–orbit peaks of Ni 2p_1/2_ and Ni 2p_3/2_ at 872.32 and 854 eV, with two shakeup satellite peaks at 879 and 861 eV, indicating that Ni^2+^ and Ni^3+^ co-existed in the NiO–NiFe_2_O_4_ sample.^44^ Similar peaks also appeared for the plasma-treated sample, however, the intensity of the peaks at 861 and 879 eV decreased. Therefore, it can be suggested that the plasma treatment under a reducing atmosphere modifies the Ni^2+^ to Ni^3+^ ratio. Moreover, it should be noted that the appearance of the double peak features in the Ni 2p spectrum along with their consecutive shake-up satellite peaks reveal the magnetic chemical states Ni^2+^ and Ni^3+^ on the plasma-treated catalyst surface.^45^

From the extensive catalyst characterisation, before and after plasma treatment, it is proposed that the mixture of compounds such as NiO–Fe_2_O_3_, inverse spinel NiFe_2_O_4_ and Fe_3_O_4_ assist CO_2_ conversion and CH_3_OH production. Moreover, the inverse spinel NiFe_2_O_4_, *i.e.* 15% NiO–Fe_2_O_3_ catalyst, shows CO_2_ conversion, however, it assists only CH_4_ production. Nevertheless, *in situ* surface investigation is needed to confirm the role of the various phases of Fe_2_O_3_ in the conversion of CO_2_ under plasma discharge.

## 5. Conclusions

In the present work, it was demonstrated that CH_3_OH could be synthesised using the binary metal oxide NiO–Fe_2_O_3_. The pure metal oxides, namely NiO and Fe_2_O_3_, exhibit no significant amount of CO_2_ conversion. However, for the binary mixed oxide, CO_2_ conversion and CH_3_OH production were greatly increased. We also provided evidence that the intermediate CO is adsorbed on the catalyst surface with modest energy, thus it can be easily hydrogenated to CH_3_OH. With an increase in the amount of NiO in Fe_2_O_3_, the particle size increased tremendously and decreased the CO_2_ conversion during thermal catalysis.

A synergistic effect is observed when plasma is combined with thermal heating, and this improves CO_2_ conversion. The CO_2_ conversion and CH_3_OH yield were increased by 3- and 5-fold, respectively, at a SIE of 0.43 kJ L^−1^ over the 10NF catalyst. The observed synergism is due to the generation of reactive species on the catalyst surface *via* plasma discharge, which speeds up CO_2_ conversion and CH_3_OH formation. The 10NF catalyst exhibited the best CH_3_OH production, whereas the 15NF catalyst was selective towards CH_4_. It can be proposed that a low amount of NiO mixing (<10%) leads to the RWGS reaction, yielding CO as the main product, however, a high amount of NiO mixing leads to CH_4_ formation.

We also showed that plasma treatment at 200 °C significantly reduced the NiO particle size, however, it did not affect the Fe_2_O_3_ particles. Moreover, it was demonstrated that the plasma treatment induced phase transformation at low temperatures and led to the formation of a mixture spinel structure. The catalyst with a mixed spinel structure assisted CO_2_ conversion and CH_3_OH production.

## Conflicts of interest

There are no conflicts to declare.

## Supplementary Material

RA-011-D1RA04852J-s001
