# Correction: Exploring the feasibility of liquid fuel synthesis from CO_2_ under cold plasma discharge: role of plasma discharge in binary metal oxide surface modification

**DOI:** 10.1039/d6ra90128j

**Published:** 2026-09-25

**Authors:** Nitesh Joshi, L. Sivachandiran

**Affiliations:** a Laboratory of Plasma Chemistry and Physics (LPCP), Department of Chemistry, Faculty of Engineering and Technology, SRM Institute of Science and Technology SRM Nagar, Kattankulathur Chennai-603203 India sivachal@srmist.edu.in

## Abstract

Correction for “Exploring the feasibility of liquid fuel synthesis from CO_2_ under cold plasma discharge: role of plasma discharge in binary metal oxide surface modification” by Nitesh Joshi *et al.*, *RSC Adv.*, 2021, **11**, 27757–27766, https://doi.org/10.1039/d1ra04852j.

The authors regret an error in the FESEM image in Fig. 3a (5% NiO–Fe_2_O_3_). Due to a mistake during the early stages of manuscript preparation the wrong image was used. The authors have re-syntheized the 5% NiO–Fe_2_O_3_ catalyst under the same experimental conditions and the corrected Fig. 3a is shown here. The revised figure accurately represents the material's morphology and does not affect the study's scientific conclusions. Table 1 has also been updated with the corrected EDX values. The correction does not affect the synthesis methodology, experimental conditions, plasma discharge parameters, or catalytic testing protocols reported in the original study.

**Fig. 3:**
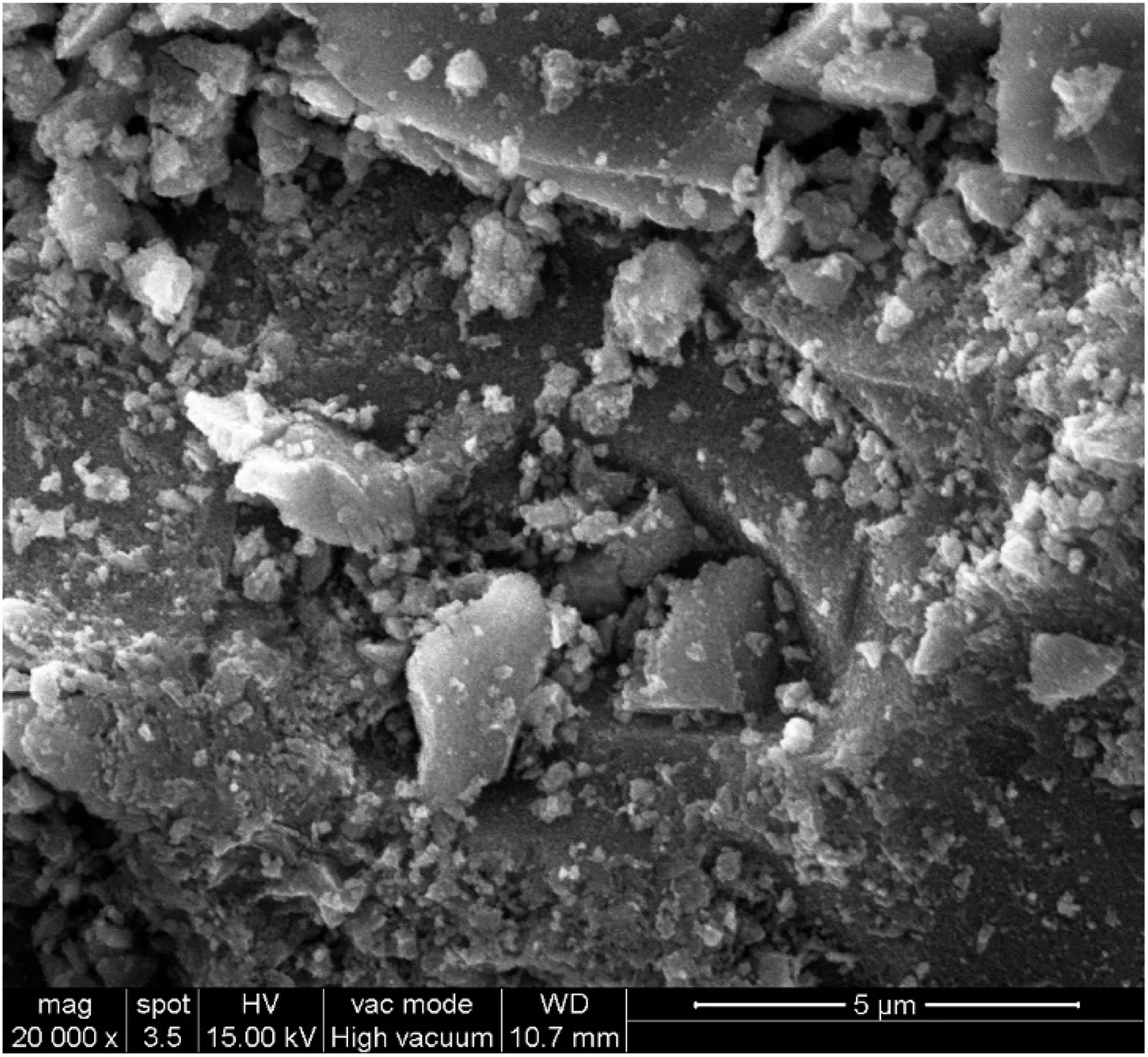
FESEM images of (a) 5% NiO–Fe_2_O_3_.

**Table 1:** Physicochemical properties of the various catalysts

Catalyst	Particle size (nm)		Metal mixing^b^ (%)	*S*_BET _ c (m^2^ g^−1^)	Pore volume (cm^3^ g^−1^)	Pore size (Å)
	NiO	Fe_2_O_3_				
Fe_2_O_3_	—	32	—	NA	NA	NA
NiO	11	—	—	NA	NA	NA
5NF	40.8	20.4	5.4	23.5	0.15	195.6
10NF	178	29.9	10.3	9.1	0.03	28.5
15NF	8934	25.7^a^	18.7	3.6	0.07	13.8

a The ICCD database reference no 00-039-1346 peak at the highest intensity at 35.631 was used.

b Results based on EDX analysis.

c The specific surface area obtained from BET measurements.

In addition, the SI has been updated to include the corrected EDX analysis corresponding to the revised data.

The authors sincerely apologize for this oversight and any inconvenience caused to the readers, reviewers, and the editorial office. The authors are committed to maintaining the highest standards of scientific integrity and transparency.

An independent expert has viewed the corrected Fig. 3a and the EDX analysis and confirmed that they are consistent with the discussions and conclusions presented.

The Royal Society of Chemistry apologises for these errors and any consequent inconvenience to authors and readers.

