# Supplementary material for: Exploring the feasibility of liquid fuel synthesis from CO2 under cold plasma discharge: role of plasma discharge in binary metal oxide surface modification
Source: RSC Adv. 2021 Aug 16;11(44):27757–66. doi: 10.1039/d1ra04852j (PMC9037809; doi:10.1039/d1ra04852j)
Supplement: RA-011-D1RA04852J-s001 [file RA-011-D1RA04852J-s001.pdf]

Electronic Supplementary Material (ESI) for RSC Advances.
This journal is © The Royal Society of Chemistry 2021

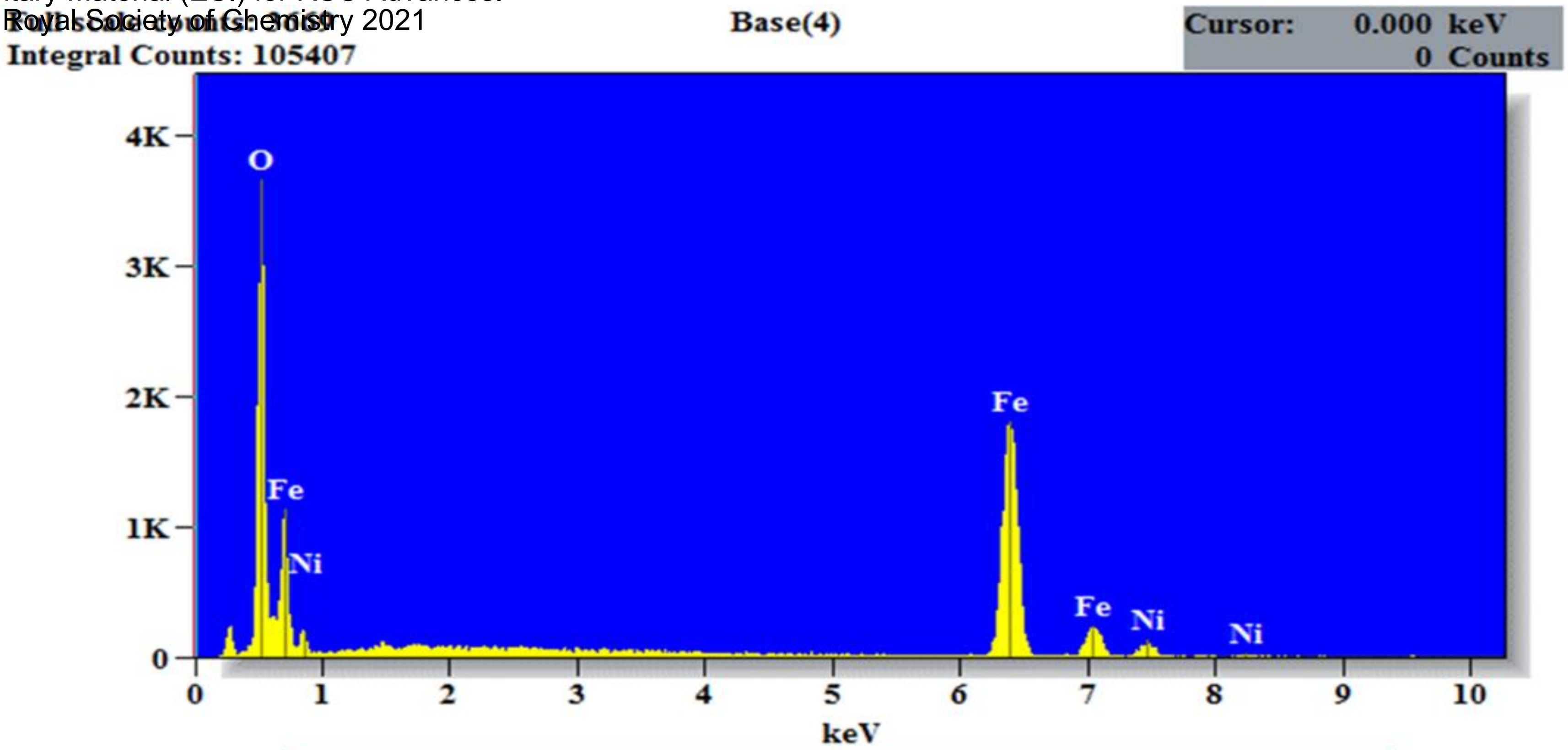

Element	Net Counts	Int. Cps/nA	Weight %	Atom %
O	19080	---	34.22	64.58
Fe	28336	---	60.57	32.74
Fe	9804	---	---	---
Ni	1645	---	5.21	2.68
Ni	1335	---	---	---
Total			100.00	100.00

Sup.Figure 1a Elemental composition for 5NF catalyst obtained using EDS

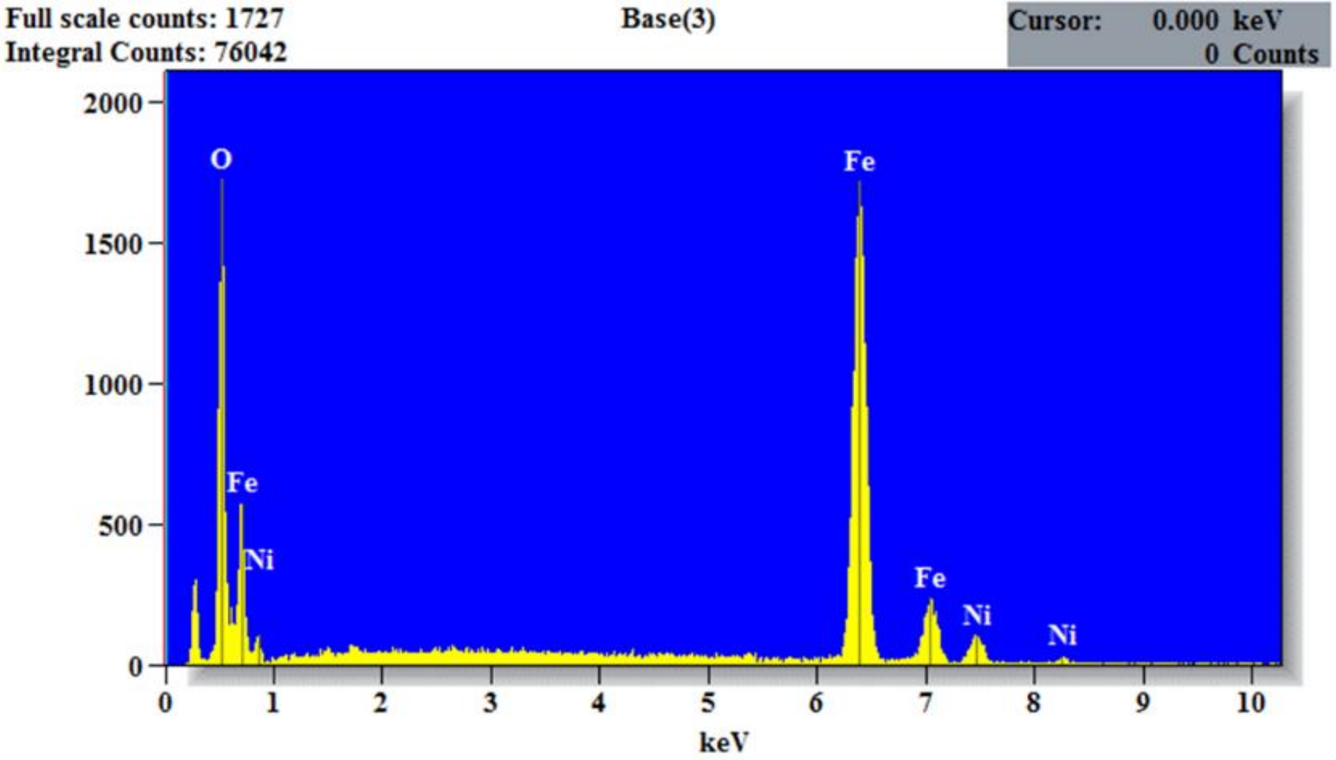

Element	Net Counts	Int. Cps/nA	Weight %	Atom %
O	8940	---	22.39	49.08
Fe	25985	---	67.31	45.84
Fe	5170	---	---	---
Ni	1545	---	10.3	5.08
Ni	719	---	---	---
Total			100.00	100.00

Sup.Figure 1b Elemental composition for 10NF catalyst obtained using EDS

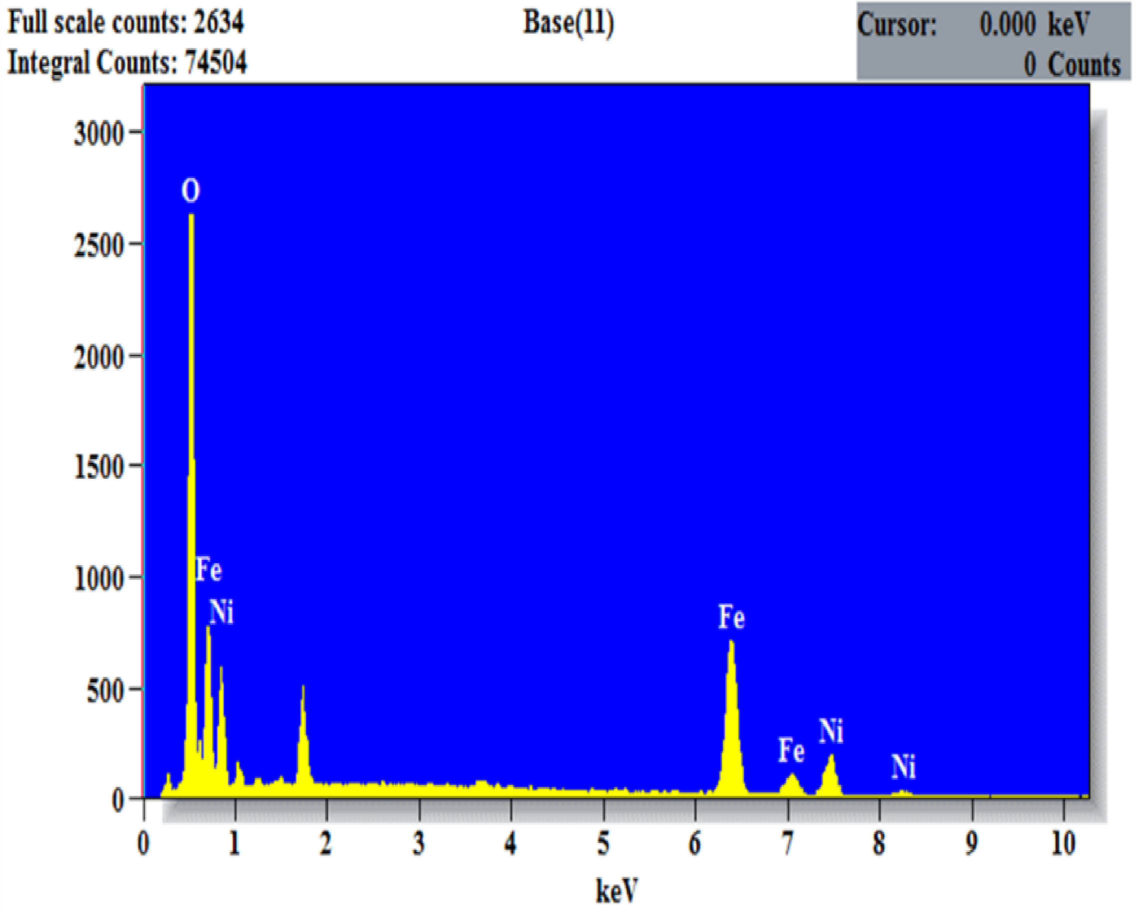

Element	Net Counts	Int. Cps/nA	Weight %	Atom %
O	14123	---	26.67	56.30
Fe	10899	---	58.03	36.27
Fe	5439	---	---	---
Ni	2732	---	15.3	7.43
Ni	3089	---	---	---
Total			100.00	100.00

Sup.Figure 1c Elemental composition for 15NF catalyst obtained using EDS

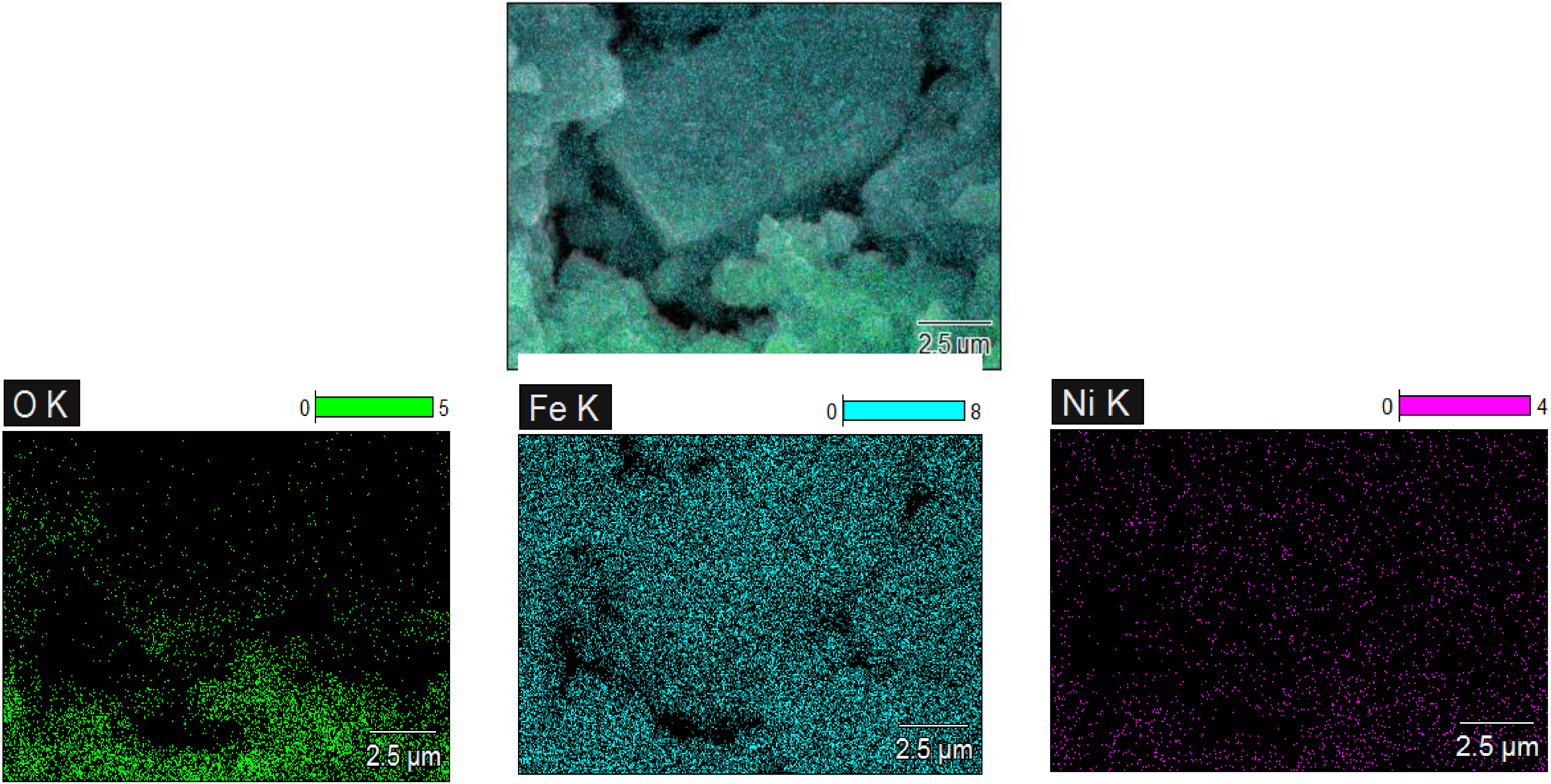

Sup.Figure 1d Elemental mapping for 10NF catalyst obtained using FESEM

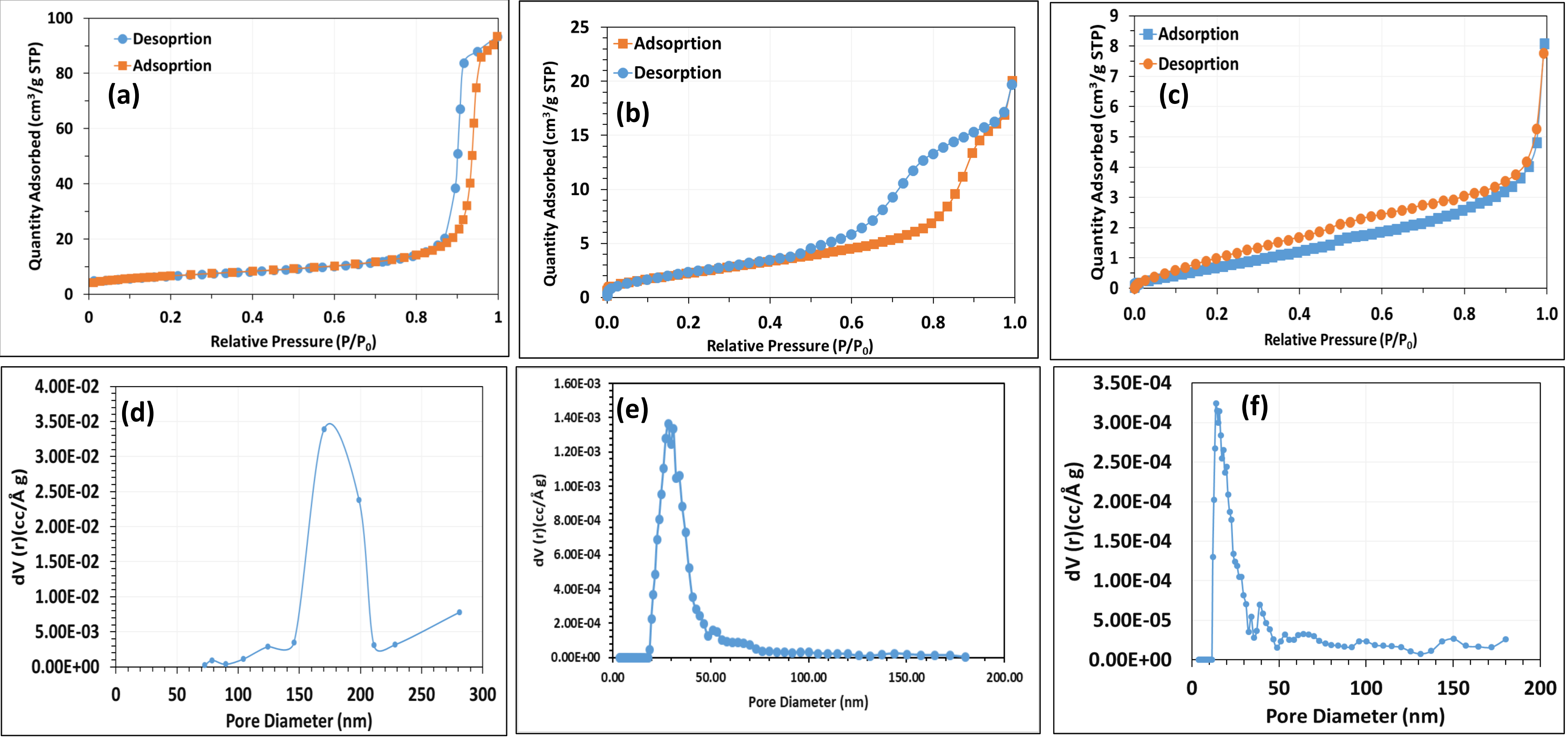

Sup.Figure 2 Adsorption-Desorption isotherm of catalysts (a) 5 NF, (b) 10 NF, (c)15 NF, (d) dV(r) vs pore diameter for 5 NF, (e) dV(r) vs pore diameter for 10 NF,(f) dV(r) vs pore diameter for 15 NF.

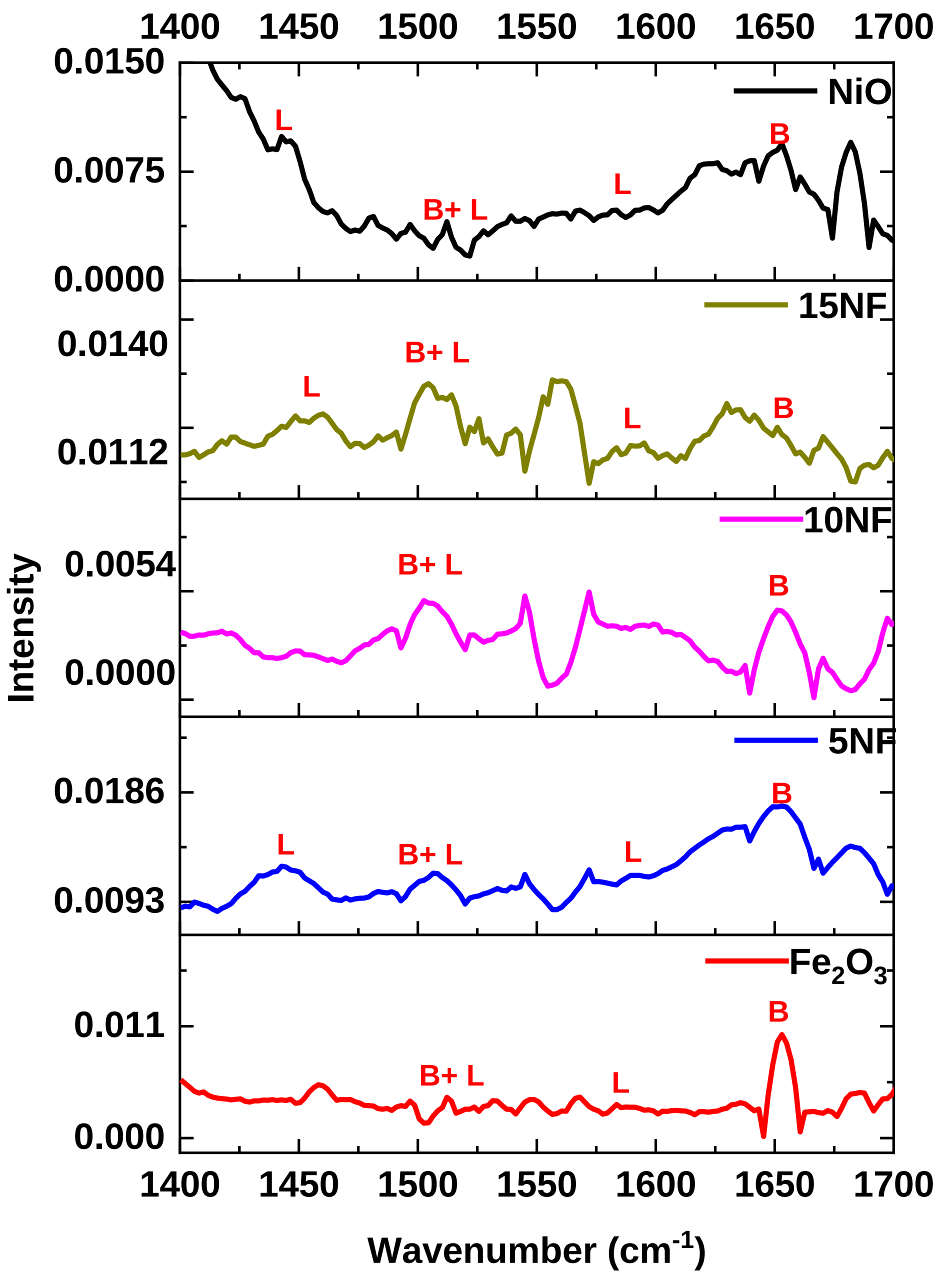

Sup.Figure 3 Pyridine adsorbed -FTIR spectra of prepared catalysts.

$$\mathbf{CO_2\ conversion}\ (\%) = \frac{[\mathbf{CO_{2(in)} - CO_{2[out]}}]}{[\mathbf{CO_{2(in)}}]} \times \mathbf{100}$$ Sup. Equation 1

$$\mathbf{CO\ selectivity}\ (\%) = \frac{[\mathbf{CO}]}{[\mathbf{CO_{2(in)} - CO_{2[out]}}]} \times \mathbf{100}$$ Sup. Equation 2

$$\mathbf{CH_4\ selectivity}\ (\%) = \frac{[\mathbf{CH_4}]}{[\mathbf{CO_{2(in)} - CO_{2[out]}}]} \times \mathbf{100}$$ Sup. Equation 3

$$\mathbf{CH_3OH\ selectivity\ of}\ (\%) = \frac{[\mathbf{CH_3OH}]}{[\mathbf{CO_{2(in)} - CO_{2[out]}}]} \times \mathbf{100}$$ Sup. Equation 4

$$\boldsymbol{Yield}\left(\boldsymbol{CH_3OH}(\%), \boldsymbol{CO}(\%), \boldsymbol{CH_{4(\%)}}\right) = \frac{[(\mathbf{S_{CH_3OH(\%)}}, \mathbf{S_{CO(\%)}}, \mathbf{S_{CH_4(\%)}}) \times \boldsymbol{X_{CO_2}}(\%)]}{\mathbf{100}}$$ Sup. Equation 5

$$\mathbf{Specific\ input\ energy(SIE\ J/L)} = \frac{\boldsymbol{Power\ injected\ into\ the\ reactor}\ (\boldsymbol{W})}{\boldsymbol{Feed\ flow\ rate}\ (\frac{\boldsymbol{L}}{\boldsymbol{S}})}$$ Sup. Equation 6

Where S_{CH_3OH} and X_{CO_2} are CH_3OH selectivity and CO_2 conversion, respectively.

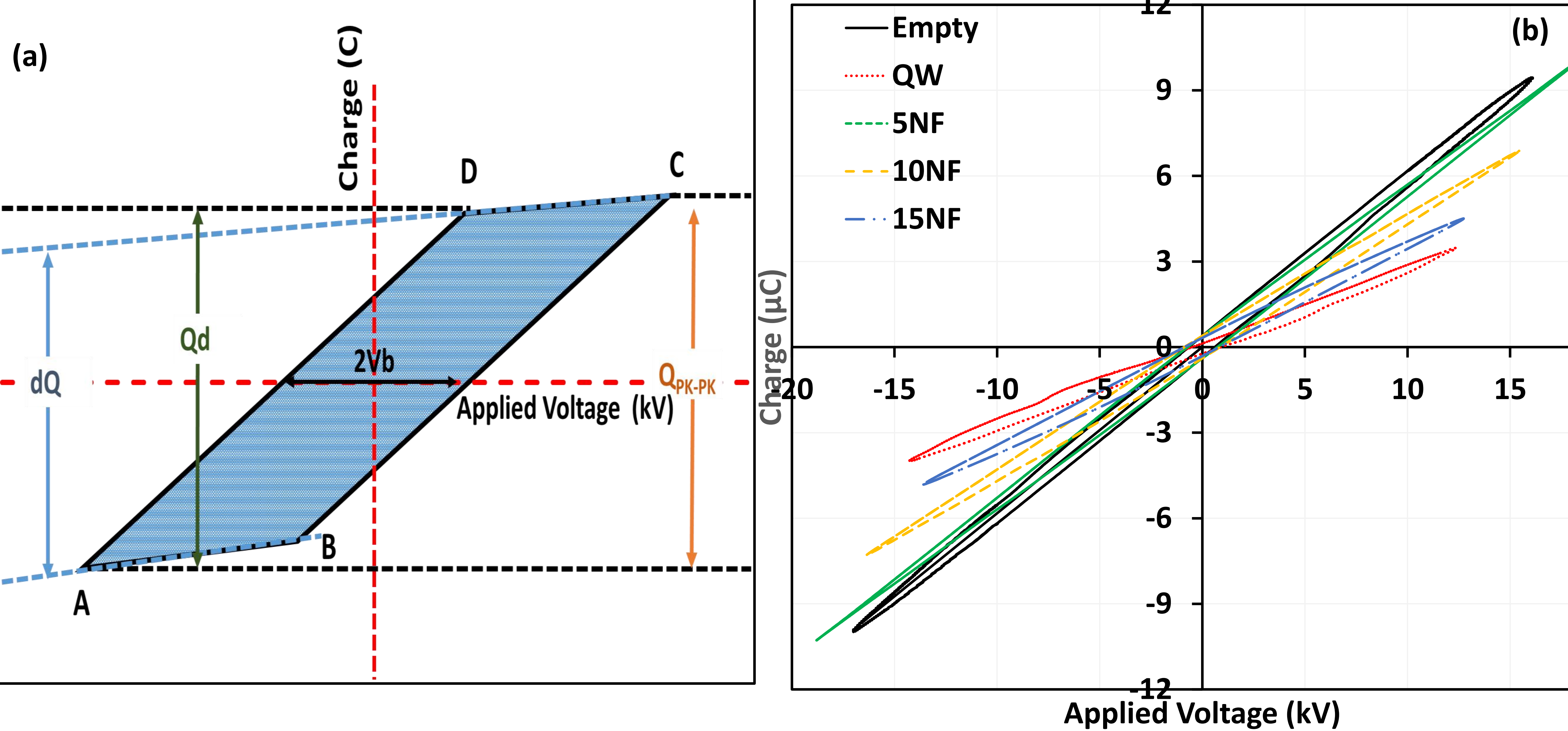

Sup. Figure 4 a) Typical schematic of Lissajous Figure, b) Discharge characteristics of reactor Lissajous figure at 14 kV for different packing materials (feed flow rate 100 ml/min, CO_2:H_2=1:3, frequency 50 Hz.)

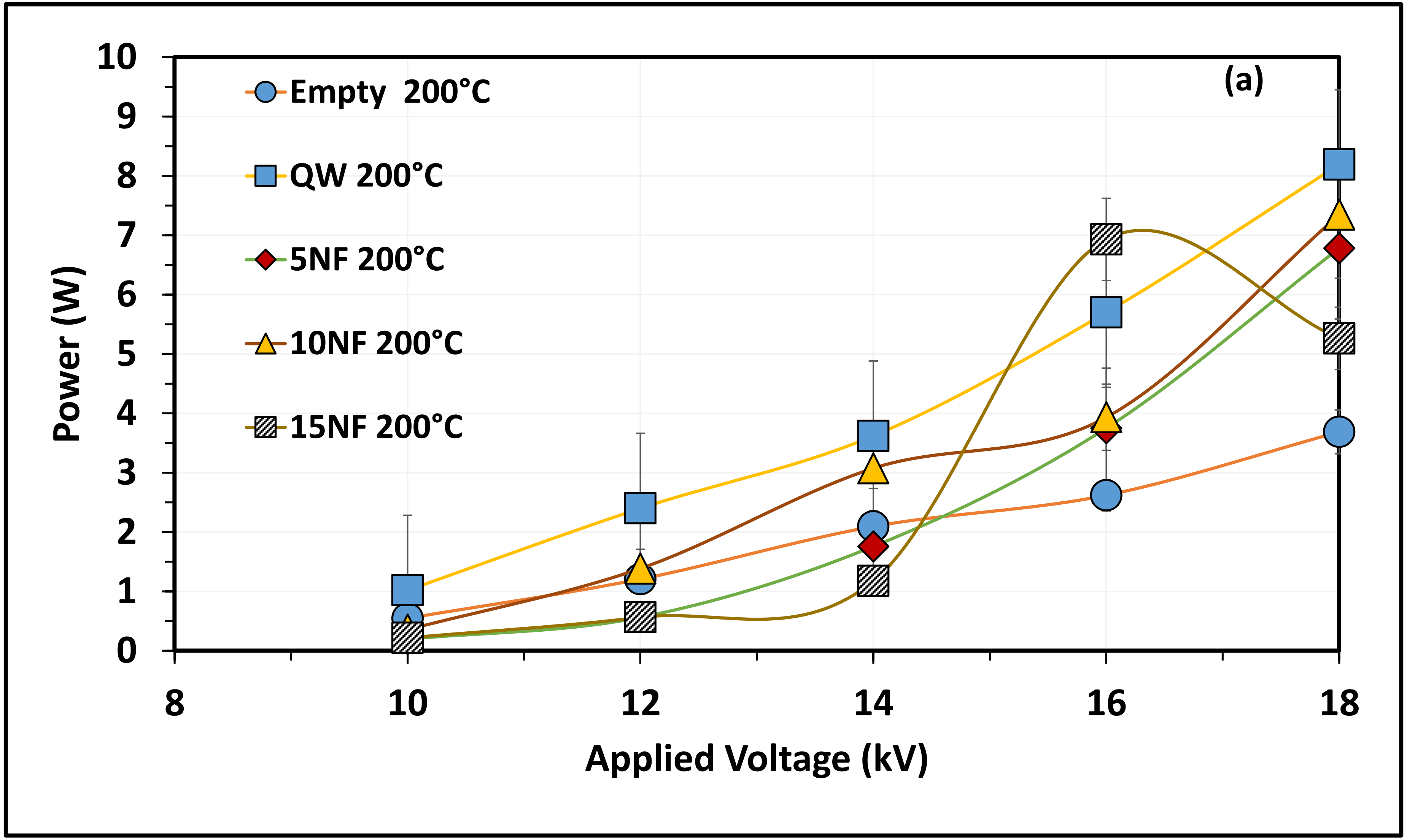

Sup.Figure 5 Discharge characteristics for various reactors: (a) impact of applied voltage on power injected into reactor ,(fee[illegible] flow rate 100 ml/min, CO_2:H_2=1:3, frequency 50 hz.)

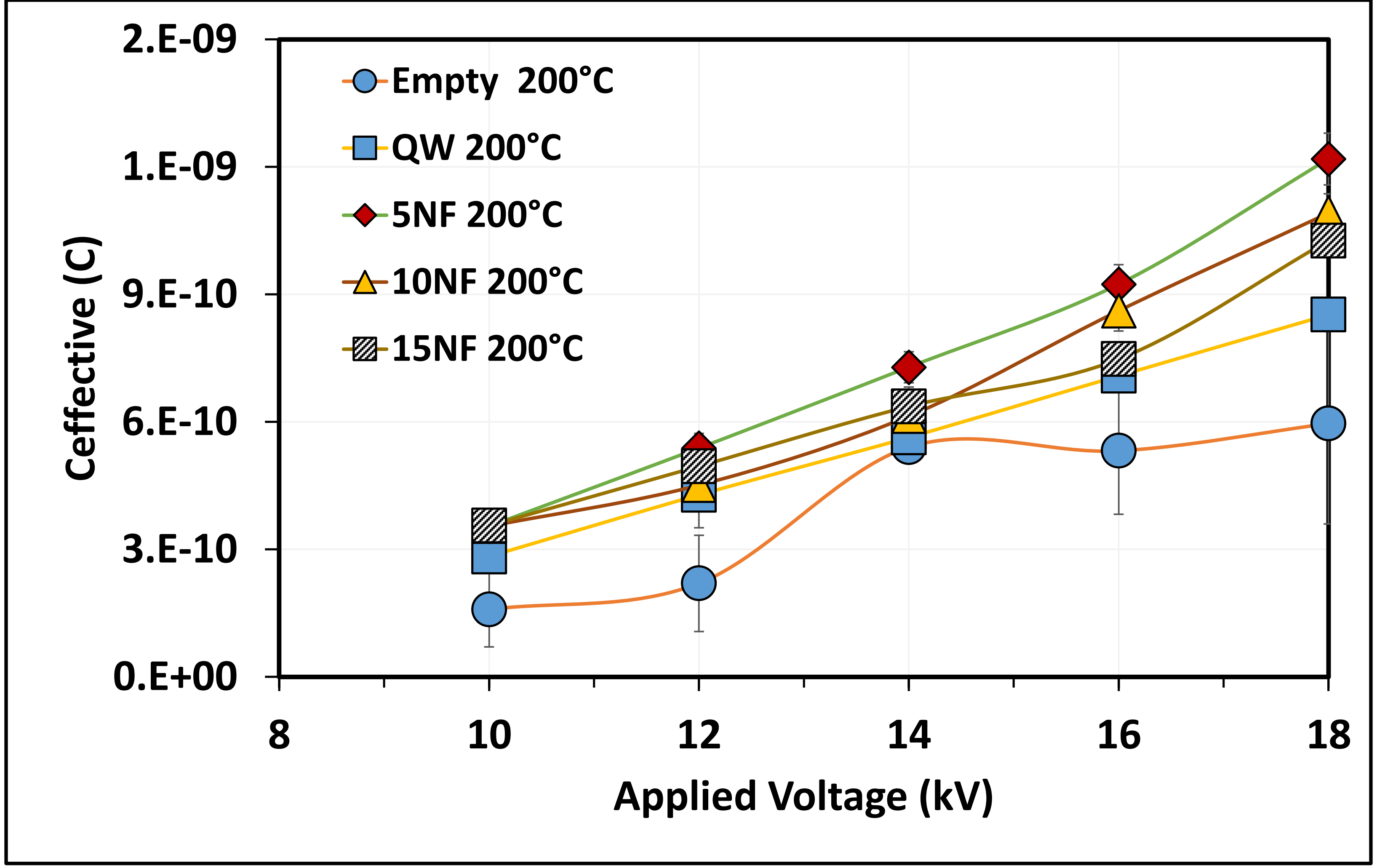

Sup.Figure 6 Discharge characteristics for various reactors: variation in effective capacitance with applied voltage, (feed flow rate 100 ml/min, CO_2:H_2=1:3, frequency 50 hz.)

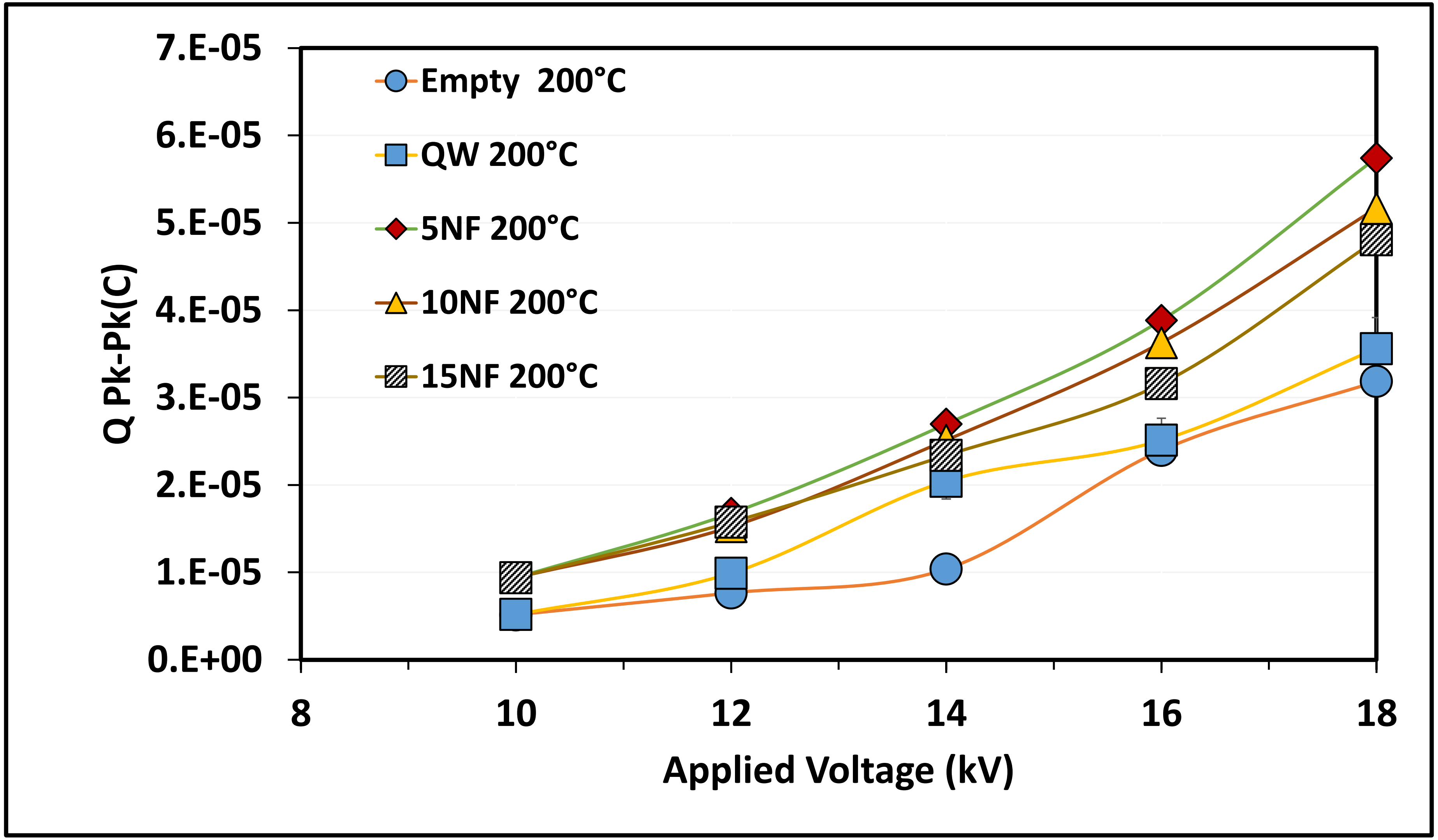

Sup.Figure 7 **Discharge characteristics for various reactors: peak charge transfer as a function of applied voltage (feed flow rate 100 ml/min, CO_2:H_2=1:3, frequency 50 hz.)**

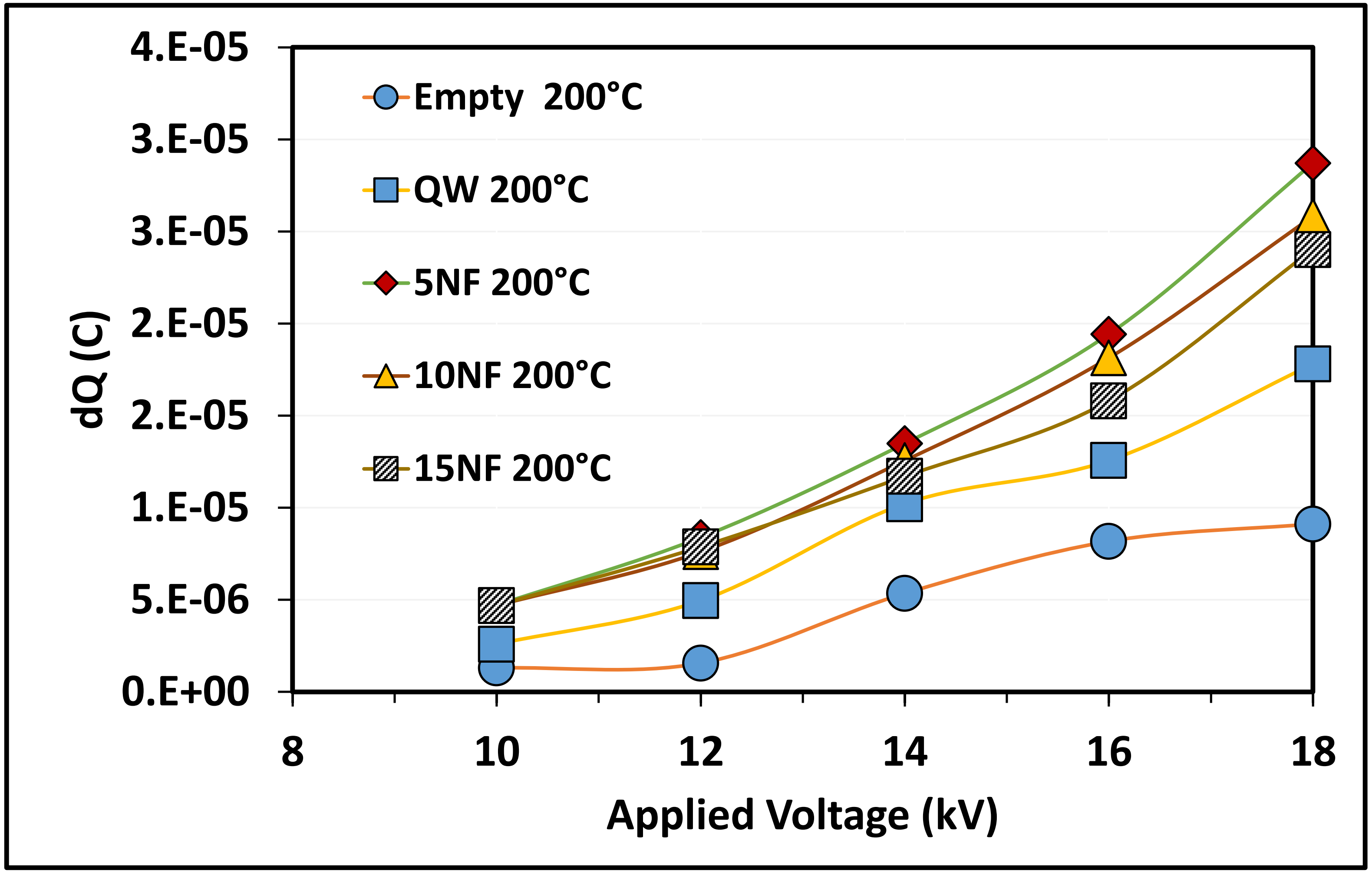

Sup.Figure 8 Discharge characteristics for various reactors: charge transfer per half cycle (feed flow rate 100 ml/min, CO_2:H_2=1:3, frequency 50 hz.)
